# An immunoinformatics-driven multiepitope subunit vaccine targeting Oropouche virus: Molecular docking, immune and dynamics simulation approaches

**DOI:** 10.1371/journal.pone.0360186

**Published:** 2026-10-09

**Authors:** Jwel Sharma, Ahmad Abdullah Mahdeen

**Affiliations:** 1 Department of Chemistry, Savitribai Phule Pune University (formerly University of Pune), Pune, India; 2 Department of Microbiology, Notre Dame University Bangladesh (NDUB), Dhaka, Bangladesh; Bangladesh Council of Scientific and Industrial Research, BANGLADESH

## Abstract

The Oropouche virus (OROV) is an emerging zoonotic orthobunyavirus responsible for recurring outbreaks of febrile illness and neurological disorders in South and Central America. Despite its growing public health issues, no FDA-approved vaccines or antiviral therapies currently exist. To address this gap, a multiepitope subunit vaccine was designed to target OROV glycoproteins G1 (Gn), G2 (Gc), and the nucleocapsid (N) protein using a reverse-vaccinology (RV) approach. For vaccine construction, suitable epitopes (MHC-I, MHC-II, and linear B-cell) were selected based on percentile rank, antigenicity, allergenicity, and toxicity. However, the biophysical attributes demonstrated advantageous characteristics, including stability and solubility (GRAVY score: −0.432). The structural modelling exhibited a Ramachandran and Z scores of 91.86% (most favoured regions) and −1.81, respectively. However, the molecular docking with TLR-2 and TLR-4 receptors showed probable interactions (−1126.8 kJ/mol) and (−1239.4 kJ/mol), respectively. Moreover, dynamics simulation of the vaccine and Vaccine-TLR-4 complexes confirmed probable dynamic stability and compactness. The codon optimisation for *Escherichia coli* K12 expression yielded a CAI value of 1.0 and a GC content of (30–70) %, indicating optimal expression. The immune simulation predicted strong antibody-mediated and T cell-mediated immunity, including elevated IFN-γ, IgG, and memory cell populations. These findings suggest the potential of multiepitope subunit vaccines as a promising preventive candidate against OROV. However, additional experimental verification is required to confirm its immunogenicity and safety in experimental settings.

## 1. Introduction

Oropouche is a re-emerging arboviral disease caused by the Oropouche virus (OROV), which belongs to the *Orthobunyavirus* genus within the *Peribunyaviridae* family. OROV was first found in Trinidad in 1955. It is a negative-sense, single-stranded RNA virus with an envelope. Its genome is made up of three segments: S, M, and L. It is related to the Iquitos and Perdido viruses in terms of its phylogeny [1,2]. These segments code for structural (S) and nonstructural proteins (NS) that are needed for the virus to replicate and hide from the immune system. People get infected mostly through the bites of infected *Culicoides paraensis midges* and *Culex* mosquitoes (*Culex quinquefasciatus*, *Coquillettidia venezuelensis*, and *Aedes serratus*), especially in the tropical rainforests of South America and the Caribbean since late 2023. In some cases, transmission can also happen through physical contact [3]. OROV infection usually shows up as Oropouche fever, which is a sudden rise in temperature (39°C) with headaches, muscle pain, joint pain, and sensitivity to light. Most infections go away on their own, but severe cases can cause neurological problems like meningitis and encephalitis, which is very bad for public health, especially in kids and the elderly. The virus is mostly thought to not be deadly, but some people may have long-lasting symptoms like vertigo, dizziness, and neurological problems [4–6]. Over 50 million cases have been reported in Brazil alone, mostly in the Amazon valley and cities. The virus seems to have spread widely, and millions of people in Latin America are expected to be affected. Even though OROV doesn’t have a very high death rate, it is becoming more common, and not reporting makes it hard to get accurate estimates of prevalence. The environment and cutting down trees make it more likely that the virus will spread to areas where it already exists [7]. Thousands of people in Brazil, Peru, Trinidad and Tobago, Panama, and other parts of South America have been affected by outbreaks. In Brazil, there were only 833 cases reported in 2023 and 13,793 cases reported in 2024. After that, RT-PCR was used to find more than 25,000 cases in thirteen South American countries between 2024 and 2025. The death rate for cases is 0.02%, and five people died between 2024 and 2025 [5]. When someone has an OROV infection, they can get chikungunya, dengue, or other arboviral diseases that cause fever. High mutation rates, problems with vaccine delivery, and different immune responses have been seen in both preclinical and clinical trials [8–10]. OROV copies itself in the cytoplasm of different types of host cells, like neurons, hepatocytes, and epithelial cells, soon after carriers carry it into the bloodstream. The viral RNA-dependent RNA polymerase (RdRp) starts the processes of transcription and replication in the RNA. OROV starts the death process in neurons by activating microglia and encouraging astrocyte activation, but these cells are not directly involved in neuronal injury. The NSs protein also lowers the host’s interferon reaction, which helps the virus avoid the immune system and replicate itself over time [11–13].

The L section codes for the RdRp enzyme, which is necessary for copying DNA. Gn, Gc, and NS are glycoproteins that are coded for by the M segment. Gn and Gc are transmembrane glycoproteins that help viruses enter cells, start to grow, and interact with immune systems. They start out as polyprotein intermediates that are broken up and controlled in the ER-Golgi complex.

However, the NS possibly participates in virion formation. The S segment encodes the nucleocapsid (N) protein along with NS, which are interferon antagonists. The N protein binds viral RNA, building the ribonucleoprotein complex essential to genome protection and efficient replication [14–16].

Several vaccine platforms exist for pathogens and cancers. Among these, live-attenuated vaccines elicit high and lasting immunity; nonetheless, they may be less suitable for immunocompromised individuals. Inactivated vaccines may be safer, although they occasionally require several boosters. Toxoid vaccines neutralise bacterial toxins rather than being ineffective against OROV. Viral vector vaccines can elicit robust immune responses; however, they can also elicit pre-existing immunity to the vector. Each platform involves trade-offs between immunogenicity, safety, and manufacturing time. Notably, multiepitope subunit vaccines (including polysaccharide, conjugate, and protein-based varieties) are even safer and elicit a strong immune response with the presence of adjuvants [17].

Reverse vaccinology (RV) and immunoinformatics have revolutionised the multiepitope subunit vaccine with rapid antigen identification, structural prediction of proteins and simulations. These *in-silico* approaches also facilitate rapid screening of the viral proteome for exceptionally stable, non-allergenic, and immunogenic epitopes without needing to culture the pathogen. This strategy has accelerated vaccine development for pathogens such as SARS-CoV-2, *Neisseria meningitidis*, *Mycobacterium Tuberculosis* (TB), ZIKA [18,19].

Despite its rising severity, there is no licensed or Food and Drug Administration (FDA)- endoresd vaccine for OROV. Therefore, this study implemented RV strategies to engineer a multi-epitope subunit vaccine targeting OROV.

## 2. Materials and methods

### 2.1 Access of targeted amino acid sequence

The polyprotein was selected (accession number NP_982303.1) from the National Center for Biotechnology Information (NCBI) (https://www.ncbi.nlm.nih.gov/protein) [20]. Subsequently, the FASTA files for the G1 and G2 glycoproteins were downloaded. The nucleocapsid (N) protein (accession number: XCO59387.1) structure was also retrieved from the same database [21]. These retrieved protein sequences were used in the development of the Oropouche virus vaccine process.

### 2.2 Prediction of major histocompatibility complex-I (MHC-I) epitopes

The Immune Epitope Database (IEDB) (http://tools.immuneepitope.org/mhci/) was utilised to assess the MHC-I epitopes from the protein sequences (G1, G2 and N) of the OROV. Human allele references were used to evaluate the MHC-I binding epitopes [22]. The projected epitopes were implemented to measure antigenicity, allergenicity, and toxigenicity through VaxiJen v2.0 (http://www.ddg-pharmfac.net/vaxijen/VaxiJen/VaxiJen.html), AllerTOP v.2.0 (https://www.ddg-pharmfac.net/AllerTOP/index.html), and ToxinPred (https://webs.iiitd.edu.in/ragha va/toxinpred/algo.php), respectively [22,23]. The parameters were kept at their default values for all servers while identifying the properties.

### 2.3 Prediction of major histocompatibility complex-II (MHC-II) epitopes

The IEDB (http://tools.iedb.org/mhcii/) web server was utilised for predicting the MHC-II binding epitopes from the targeted proteins (G1, G2 and N). The predicted epitopes were then used to assess antigenicity, allergenicity, and toxigenicity using the aforementioned servers. Only epitopes that met all three criteria were retained for further inclusion in vaccine construction [24,25]

### 2.4 Linear B-cell epitope prediction

The linear B-cell epitopes from the targeted proteins (G1, G2 and N) were identified by the ABCpred (https://webs.iiitd.edu.in/raghava/abcpred/) server. The ABCpred server can predict the epitopes from a specific protein [26]. However, ABCpred uses a recurrent neural network algorithm with an accuracy rate of 65.93% [24,25]. Subsequently, the identified linear B-cell epitopes were implemented to examine all the selected criteria, including antigenicity, allergenicity, and toxigenicity, using the aforementioned servers. All the parameters were kept at their defaults during the checking of the required criteria.

### 2.5 Construction of the candidate

The best ranked peptides (epitopes) of G1, G2 and N proteins were employed to design the vaccine. To enhance immunogenicity, an adjuvant (50S ribosomal protein of *M. tuberculosis)* was incorporated at the N-terminal of the vaccine [27]. The adjuvant was deliberately placed at the N-terminal region, as this configuration is frequently utilised in multi-epitope vaccine development and is confirmed by immunoinformatics research. Positioning the adjuvant at the N-terminus enables its first translation and maintains its structural differentiation from the subsequent epitope sections, thus promoting effective identification by pattern recognition receptors (PRRs), including Toll-like receptors, contingent upon the chosen adjuvant. This orientation is anticipated to augment the primary innate immune activation, hence fostering more robust adaptive immunological reactions. Furthermore, the positioning of the N-terminal, in conjunction with a suitable stiff linker (such as EAAAK), aids in reducing structural disruption between the adjuvant and the epitope sequences, hence maintaining the autonomous folding and biological efficacy of each element. However, this is not an established immunological advantage rather than previously utilized strategy in similar type of researches. The linkers were used to ensure proper separation and folding: EAAAK was utilised between adjuvant and MHC-I epitopes, while KKK linkers were used between subsequent epitopes. Additionally, a 6xHis tag (HHHHHH) was added at the C-terminal for purification [28].

### 2.6 Demographic coverage analysis

The expression of (Human Leukocyte Antigen) HLA alleles changes by location and ethnic groups [29,30]. The IEDB server (http://tools.iedb.org/population/) was implemented to measure the efficacy of the OROV vaccine among various individuals [31]. Subsequently, selected “MHC-I” and “MHC-II” epitopes, together with their associated HLA alleles, were appointed both individually and collectively across several continents.

### 2.7 Assessment of biophysical entities

The biophysical entities of the vaccine were measured with the Expasy ProtParam server (http://web.expasy.org/protparam/) [32]. This analysis evaluates the aliphatic index, the overall scale of atoms, the isoelectric point (pI), the grand average of hydropathicity (GRAVY), molecular weight, instability, and the total number of amino acids. However, the solubility of the vaccine was predicted using SOLpro (https://scratch.proteomics.ics.uci.edu/) server, and its overexpression behaviour was analysed to ensure optimal performance of the host system [33].

Moreover, the allergenicity of the vaccine was determined with AllergenFP v.1.0 (https://ddg-pharmfac.net/AllergenFP/), AllerTOP v.2.0 (https://www.ddg-pharmfac.net/AllerTOP/) servers [34]. Then, ANTIGENpro (http://scratch.proteomics.ics.uci.edu) and VaxiJen 2.0 (http://www.ddg-pharmfac.net/vax ijen/VaxiJen/VaxiJen.html) were employed to determine the antigenicity of the vaccine [22,35]. All the necessary parameters were kept as the default.

### 2.8 Prediction of secondary model

The secondary modelling of the vaccine was determined using GOR4 (https://npsa-pbil.ibcp.fr/cgi-bin/npsa_automat.pl?page=/NPSA/npsa_gor4.html), SOPMA (https://npsa-prabi.ibcp.fr/cgi-bin/npsa_automat.pl?page=/NPSA/npsa_sopma.html), and the PSIPRED (http://bioinf.cs.ucl.ac.uk/psipred/) [36]. The GOR4 server predicts the secondary model of the vaccine based on Bayesian statistical analysis and information theory. Furthermore, the PSIPRED server uses two feedforward neural networks to produce structural outputs from PSI–BLAST. However, the SOPMA server determines the highest quantity of amino acids by a ternary illustration, such as beta sheet, alpha-helix, and coil structure.

### 2.9 Modelling, improvement, validation, and energy minimisation of tertiary model

The candidate was modelled using the I-TASSER server (https://zhanggroup.org/I-TASSER/) [37]. While the prediction procedure, I-TASSER server employs several threading alignments and sequential simulations of template fragment assembly, which normalise the tertiary (3D) structure of a predicted protein [38]. The Disulfide by Design 2 v2.13 server (http://cptweb.cpt.wayne.edu/DbD2/) was utilized to ascertain the possible cysteine disulfide bonding pairs inside the vaccine construction [39]. The server identifies the probable sites within the protein’s structure that are more probable to form disulfide bonds. The selected pair of residues for mutation was based on its low energy and χ3 angle, which spans a broad range of − 87° or + 97° ± 30 [39]. Later, the 3D was improved utilizing the GalaxyWEB server (https://galaxy.seoklab.org/cgi-bin/submit.cgi?type=REFINE) [40]. Then, the SAVES v6.0 server (https://saves.mbi.ucla.edu/) was used to identify additional structural validation of the vaccine, which weighs the vaccine model’s stereochemical quality [41–43]. Additionally, the model’s overall quality was confirmed using the ProSA (https://prosa.services.came.sbg.ac.at/prosa.php) and Ramplot (https://www.ramplot.in/) tools, which calculates the Z-score to assess correctness and identify potential errors and Ramachandran score to assess protein quality, respectively [44,45].

### 2.10 Structural B-cell epitopes prediction

To determine the structural B-cell epitopes, the Ellipro (https://tools.iedb.org/ellipro/) was employed [46]. The default settings were used for the prediction.

### 2.11 Molecular docking analysis

The molecular docking was conducted utilising the Cluspro 2.0 (https://cluspro.bu.edu/login.php) server with Toll-like Receptors (TLRs) and the vaccine candidate to scrutinize the molecular-level interactions [47,48]. For the analysis, the 3D models of human TLR-2 (“Toll-like receptor 2”) (PDB: 2Z7X) and human TLR-4 (“Toll-like receptor 4”) (PDB: 3FXI) were downloaded from the PDB server (https://www.rcsb.org/). Subsequently, the docked complexes were viewed and examined through PDBsum (https://www.ebi.ac.uk/thornton-srv/databases/pdbsum/Generate.html) and PyMOL (https://pymol.org/2/) [49]. Both receptors can readily detect PAMPs (pathogen-associated molecular patterns) and promote both innate and adaptive immune responses [50].

### 2.12 Calculation of free energy via molecular mechanics with generalized born and surface area solvation (MM-GBSA)

The HawkDock (https://cadd.zju.edu.cn/hawkdock/) server was employed for conducting the MM-GBSA (Molecular Mechanics Generalized Born Surface Area) to ascertain the free energy involved in Vaccine-TLR-2 and Vaccine-TLR-2 compounds [51,52]. Various molecular mechanics were being evaluated, such as binding contact analysis, Van der Waals forces (VDW), polar (GB), electrostatic interaction (ELE), and non-polar (SA) components.

### 2.13 Normal mode analysis

Normal Mode Analysis was conducted by utilizing the iMOD (https://imods.chaconlab.org/) web server [53,54]. The server primarily assesses the stability and dynamics of the vaccine’s molecules and atoms. The analysis provides the conformational alterations and atomic-level interactions between “TLR-2” and “TLR-4” in the presence of the vaccine. These features support the vaccine’s conformational stability and potential efficiency. The parameters were set to default settings.

### 2.14 Molecular dynamics simulation

The molecular dynamics simulation of the apo (vaccine model) and the Vaccine-TLR-4 complex was performed utilizing the Groningen Machine for Chemical Simulations (GROMACS) (version 2022.3) software [55]. A triclinic water box employing the SPC water model and the GROMOS96 43a1 force field was utilized for this simulation [56]. Furthermore, all necessary ions were employed to equilibrate the system. Following the establishment of equilibrium under isothermal-isochoric (NVT) and isobaric (NPT) conditions, and subsequent energy minimization, a molecular dynamics simulation lasting 100 nanoseconds (ns) was conducted utilizing periodic boundary conditions and a time integration step of 2 femtoseconds (fs). The trajectory data was evaluated at a temporal resolution of 100 picoseconds (ps). Nonetheless, GROMACS computed the root mean square deviation (RMSD), root mean square fluctuations (RMSF), radius of gyration (Rg), solvent accessible surface area (SASA), and hydrogen bond (H-bond) assessment. Furthermore, the ggplot2 software in RStudio visually depicted each individual analysis [57].

### 2.15 Codon optimization and computational cloning of vaccine

To conduct computational cloning and optimization of codon, the JCat (https://www.jcat.de/Start.jsp) server was used by using the *E. coli* strain K12 [58,59]. The server determines the expression level by evaluating the Codon Adaptation Index (CAI) and the guanine-cytosine (GC) composition of a synthesized protein. Subsequently, the vaccine gene sequence was incorporated into the *E. coli* plasmid vector pET-28a (+) via the Eco53kI and EcoRV restriction sites and cloning were assessed by SnapGene (https://www.snapgene.com/free-trial/) [60].

### 2.16 Immune response simulation

The immune simulation was evaluated using the C-ImmSim server (https://kraken.iac.rm.cnr.it/C-IMMSIM/) [45]. The sever predicts both the humoral and cellular-mediated immune responses. The vaccine was administered in three doses at four-week intervals prior to full vaccination. All simulation parameters retained their default values. The steps and volume were set to 1000 and 50, respectively, and the random seed was set to 12,345 by default [61].

### 2.17 Validation of the mRNA vaccine structure

The JCat server predicted DNA sequence was converted to RNA sequence by the Biomodel (https://biomodel.uah.es/en/lab/cybertory/analysis/trans.htm) server [58,62]. Subsequently, the RNAfold server (https://rna.tbi.univie.ac.at/cgi-bin/RNAWebSuite/RNAfold.cgi) determined the secondary structure of the vaccine [63]. The web server is capable of quantifying the minimal free energy (MFE) of mRNA structures. Nevertheless, the required parameters were set as default, where temperature and salt concentration were 37˚C and 1 M, respectively [35].

## 3. Results

### 3.1 Access of amino acid sequences

The polyprotein and nucleocapsid were collected from the NCBI server. Thereafter, G1 and G2 glycoproteins were retrieved from the polyprotein. The overview of the study is demonstrated in Fig 1.

**Fig 1:**
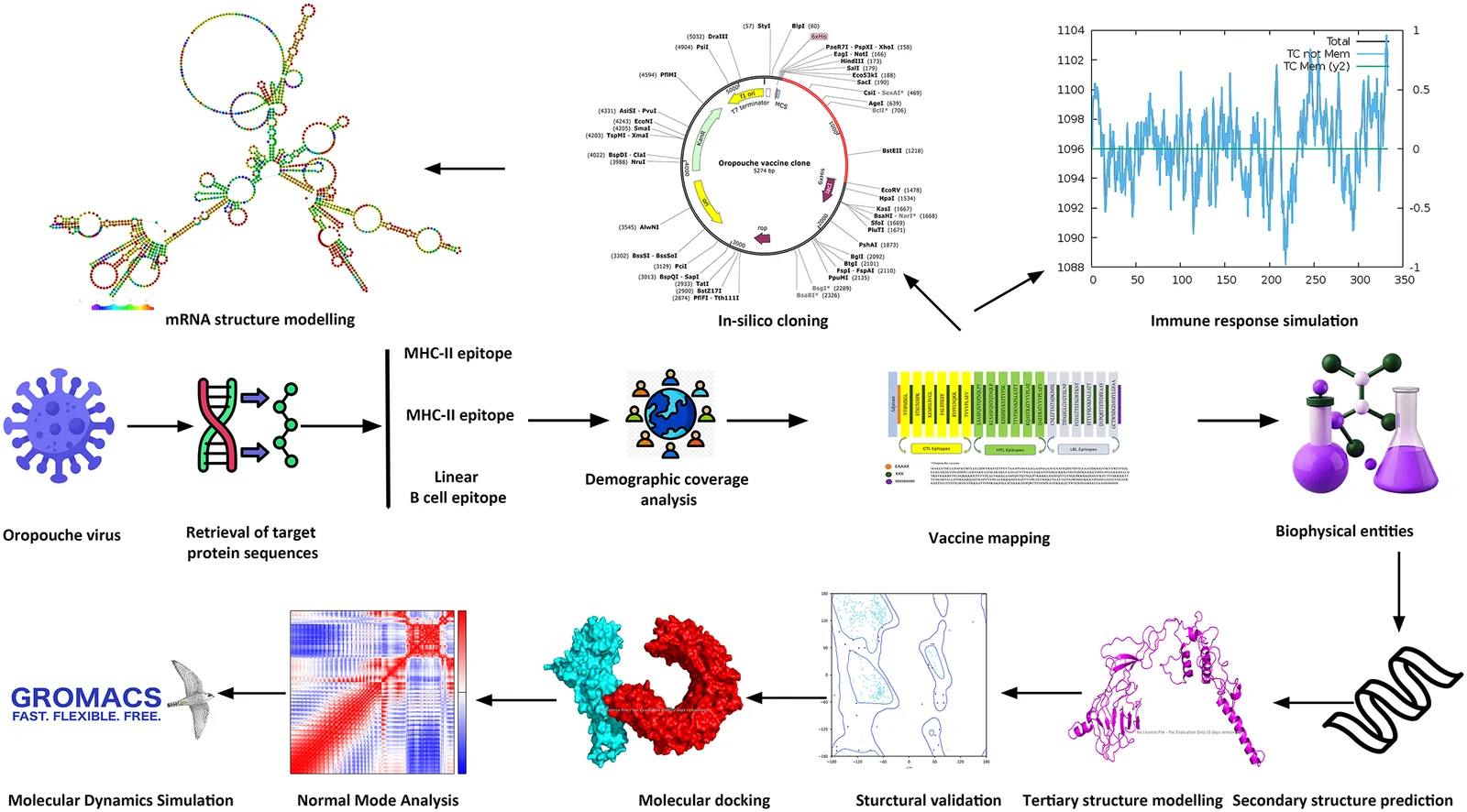
Overview of multiepitope subunit vaccine design.

### 3.2 Prediction of MHC-I epitopes

Two peptides from each protein, totalling six nine-amino-acid peptides, were selected using the IEDB web server based on percentile rank (≤1.00) and affinity score. These peptides were evaluated for antigenicity, allergenicity, and toxicity prior to future vaccine mapping. Ultimately, all six peptides that satisfied the criteria were selected for subsequent vaccine development (Table 1).

**Table 1:** The predicted MHC-I epitopes and their antigenicity, allergenicity, and toxicity.

Protein	Epitopes	Percentile rank	Antigenicity	Allele	Toxicity	Allergenicity
G1	NTIPAISGL	0.01	0.7865 (Antigenic)	27	No	No
	STIGTGSPK	0.01	1.0067 (Antigenic)	27		
G2	KEMNLSVGL	0.02	1.9084 (Antigenic)	27		
	FALIITKSY	0.02	1.0567 (Antigenic)	27		
Nucleocapsid	RVFFLNQKK	0.02	1.1076 (Antigenic)	27		
	TVVVPLAEV	0.02	0.4265 (Antigenic)	27		

### 3.3 Prediction of MHC-II epitopes

Two peptides were extracted from each polyprotein component (G1 & G2) and the Nucleocapsid, as well as six fifteen-mer peptides selected based on affinity score and percentile rank. The chosen six epitopes were evaluated for allergenicity, toxicity, and antigenicity. After satisfying all previously indicated criteria, the epitopes were deemed suitable for the final vaccine formulation (Table 2).

**Table 2:** The anticipated MHC-II epitopes together with their antigenic, allergenic, and toxic properties.

Protein	Epitopes	Percentile rank	Allele	Antigenicity	Allergenicity	Toxicity
G1	LSASFQNTQTNQLPT	0.04	27	0.6363 (Antigenic)	No	No
	KLSASFQNTQTNQLP	0.06	27	0.7326 (Antigenic)		
G2	KDDISIVKSTTVFSK	0.08	27	0.4681 (Antigenic)		
	TTVFSKNKPALEATT	0.12	27	0.4287 (Antigenic)		
Nucleocapsid	KQAEFRATVVVPLAE	0.02	27	0.7028 (Antigenic)		
	QAEFRATVVVPLAEV	0.03	27	0.8498 (Antigenic)		

### 3.4 Prediction of linear B-cell epitopes

Two peptides (antigenic, non-allergenic, and non-toxic) were obtained from each protein via the ABCpred server (Table 3).

**Table 3:** The predicted linear B-cell epitopes together with their antigenic, allergenic, and toxic properties.

Protein	Peptides	Antigenicity	Toxicity	Allergenicity	Start
G1	CNLEFTAITADKIMSL	1.0627 (Antigenic)	No	No	255
	TIPAISGLGVGYHLNF	1.1559 (Antigenic)			430
G2	FEIVGTTSTSGWFKNT	0.6525 (Antigenic)			114
	STTVFSKNKPALEATT	0.4383 (Antigenic)			32
Nucleocapsid	DVPQRTTSTFDPEAAY	0.542 (Antigenic)			8
	GCTWNDGDAMYLGFAA	0.4166 (Antigenic)			129

### 3.5 Construction of candidate

The candidate was developed using MHC-I, MHC-II, and linear B-cell epitopes derived from polyprotein (G1, G2) and nucleocapsid proteins. To achieve optimal connectivity, the EAAAK linker was used in conjunction with an adjuvant (50s ribosomal protein *M. tuberculosis*) and MHC-I epitopes. Following the remaining epitopes, the linker KKK was used (Fig 2).

**Fig 2:**
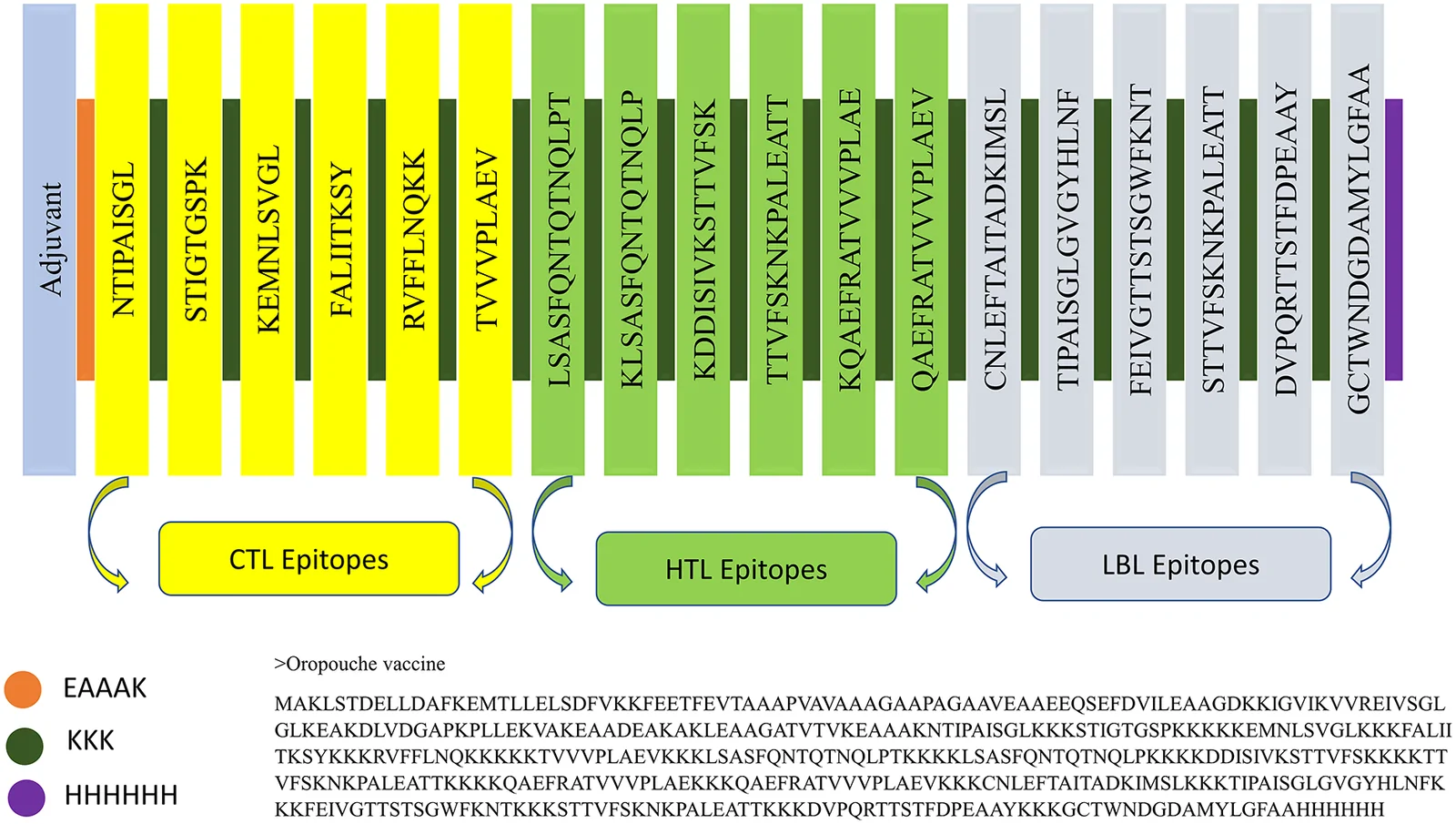
The vaccine mapping with MHC-I, MHC-II, B-cell epitopes, linkers, and an adjuvant and other essential components.

### 3.6 Demographic coverage analysis

In the demographic coverage, the MHC-I and MHC-II epitopes contributed 98.55% and 99.99%, respectively, and the predicted HLA coverage was 100%. East Asia, Northeast Asia, South Asia, Europe, East Africa, West Africa, Central America, North America, the West Indies, South America, and Oceania received the most coverage, accounting for a combined total of 100% of MHC-I and MHC-II epitopes. The three remaining regions are Southeast Asia (99.98%), Southwest Asia (99.99%), and South Africa (95.27%) (S1 Fig). Due to the utilization of the most frequent HLA alleles, the coverage was high, but it is not definitive (S1 Table).

### 3.7 Assessment of the biophysical elements of the vaccine

The ExPASy ProtParam server determined the molecular weight to be 47107.23 Da and the amino acid composition to be 432. The isoelectric point (pI) was 9.95, indicating that it was a basic protein. The GRAVY (−0.432), indicated that the vaccine was both hydrophilic and water-soluble. Moreover, the instability and aliphatic scores were 18.29 and 78.66, respectively, implying stability and thermostability. Subsequently, the SOLpro server predicted water solubility when expressed in *E. coli*, yielding a score of 0.742. Finally, allergenicity was assessed using AllerTOP v. 2.0, AllergenFP v. 1.0, and the AlgPred servers. Moreover, the antigenic properties were assessed using VaxiJen 2.0, and ANTIGENpro, respectively, confirming the vaccine’s suitability for consideration as a vaccine candidate (Table 4).

**Table 4:** Biophysical elements of the OROV vaccine.

Biophysical elements	Value
Number of amino acids	432
Molecular weight	47107.23
Theoretical pI	9.95
(-) Charged residues (Asp + Glu)	44
(+) Charged residues (Arg + Lys)	88
Formula	C_2128_H_3520_N_570_O_612_S_7_
Amounts of atom	6837
Extinction coefficients in water	17085
Estimated half-life	>10 hours (*E. coli*, *in-vivo*)
Instability index	18.29 (stable)
Aliphatic index	78.66
GRAVY	−0.432
Predicted scaled solubility (Sol pro manchester)	0.742
pI (Sol pro)	10.39
Predicted solubility upon overexpression (Scratch)	0.95138
Allergenicity (AllerTOP v. 2.0/AllergenFP v.1.0)	Negative
Antigenicity (VaxiJen 2.0/ANTIGENpro)	Positive

### 3.8 Modelling of secondary structure

The GOR4, PSIPRED, and SOPMA servers were employed to ascertain the secondary modelling of the vaccine. The vaccine consists of alpha helix (49.49%), random coil (36.34%), and extended strand (beta sheet) (14.35%) as anticipated by GOR4. However, the SOPMA also identified alpha helix (37.37%), random coil (40.97%), and extended strand (beta sheet) (16.20%) (S2 Table). The vaccine’s secondary model, alongside its properties (alpha helix, random coil, and extended strand) was demonstrated by PSIPRED (S2 Fig).

### 3.9 Prediction and validation of tertiary model and disulfide engineering

The I-TASSER server provided five different 3D models of the OROV vaccine. Among the five models, the best was elected based on the standard C score (−1.14), RMSD (9.6 ± 4.6 Å), and TM score (0.57 ± 0.15) (Fig 3D). To enhance the stability of the predicted model, the Disulfide by Design 2 v2.13 server was utilized to implement disulfide engineering. In total, pairs of 36 amino acids were recognized as appropriate for the construction of disulfide bonds. Nonetheless, the energy and Chi3 metrics of four residue pairs fell inside the acceptable range. As a result, the residue pairs were altered with four mutations: PRO_39-ALA_41 (energy of 2.38 kcal/mol and Chi3 value of + 86.42), ALA_44-ALA_69 (Chi3 value of −106.09 and an energy of 2.26 kcal/mol), PRO_103-MET_420 (energy of 2.74 kcal/mol and Chi3 value of + 106.57) and VAL_185-GLN_235 (energy of 2.76 kcal/mol and Chi3 value of + 116.16) (S3 Fig and S3 Table). Following that the refinement of the vaccine structure was conducted by the Galaxy Web server, which provided the MolProbity score (2.449), RMSD (0.474), and Ramda favoured (89.3). The Ramachandran plot from the Ramplot server illustrated that the maximum amino acid residues occupied the most favorable regions 91.86%, allowed regions and disallowed regions 7.21% and 0.93%, respectively (Fig 3A). Subsequently, the ProSA server yielded a Z-score of −1.81 (Fig 3C). The ERRAT score of the refined vaccine structure was determined, which was 71.85 (Fig 3B).

**Fig 3:**
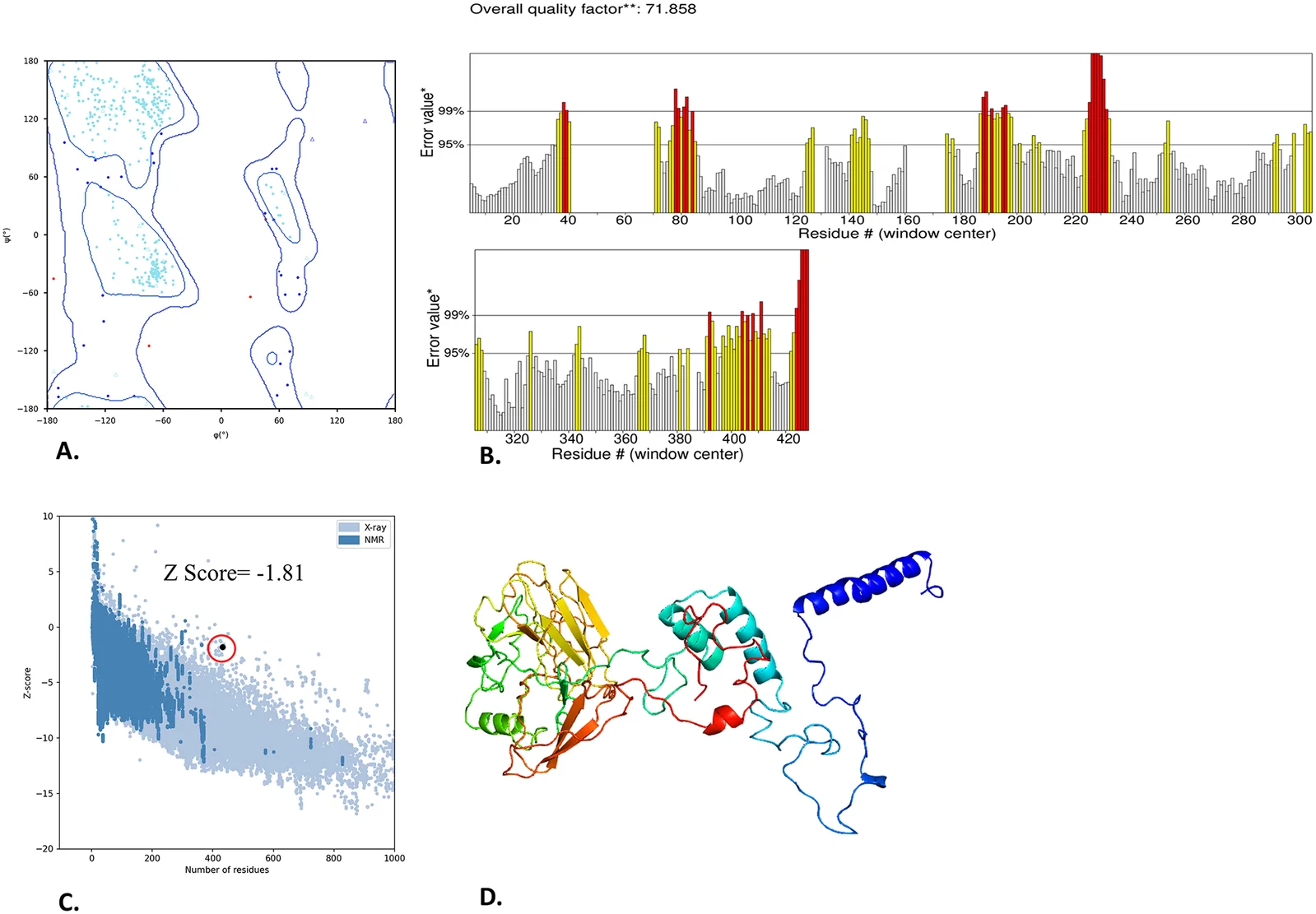
Tertiary model of OROV vaccine with (A) Ramachandran plot, (B) ERRAT, (C) Z-score and (D) Ribbon 3D model.

### 3.10 Prediction of structural B-cell epitopes

The Ellipro server identified four structural B-cell epitopes within a total of 260 residues, with scores between 0.51 and 0.72 (S4 Fig and S4 Table).

### 3.11 Molecular docking

The Cluspro 2.0 server was utilized to analyze the molecular interaction between the vaccine and human Toll-like receptors (TLRs), specifically TLR-2 and TLR-4. The server provided 40 structures, from which the Vaccine-TLR-2 complex with the lowest binding energy of −1126.8 and central energy of −873.9, as well as the Vaccine-TLR-4 complex with the lowest binding energy of −1239.4 and central energy of −1162, were elected (Table 5). Notably, the energy scores by Cluspro 2.0 do not directly estimate the binding free energy but rather are utilised for relative ranking of the most probable poses of the docked complexes. So, these scores are predictive rather than definitive. However, for visualization and molecular interactions of docked complexes, Pymol and PDBsum were employed. According to PDBsum, Vaccine-TLR-2 possessed 4 salt bridges and 35 hydrogen bonds, while Vaccine-TLR-4 contained 5 salt bridges and 28 hydrogen bonds (Fig 4 and Table 5).

**Table 5:** Docking and interaction score of Vaccine-TLRs complexes.

Vaccine complex	Hydrogen bond	Salt bridge	Lowest energy	Center
Vaccine-TLR-2	35	4	−1126.8	−873.9
Vaccine-TLR-2	28	5	−1239.4	−1162

**Fig 4:**
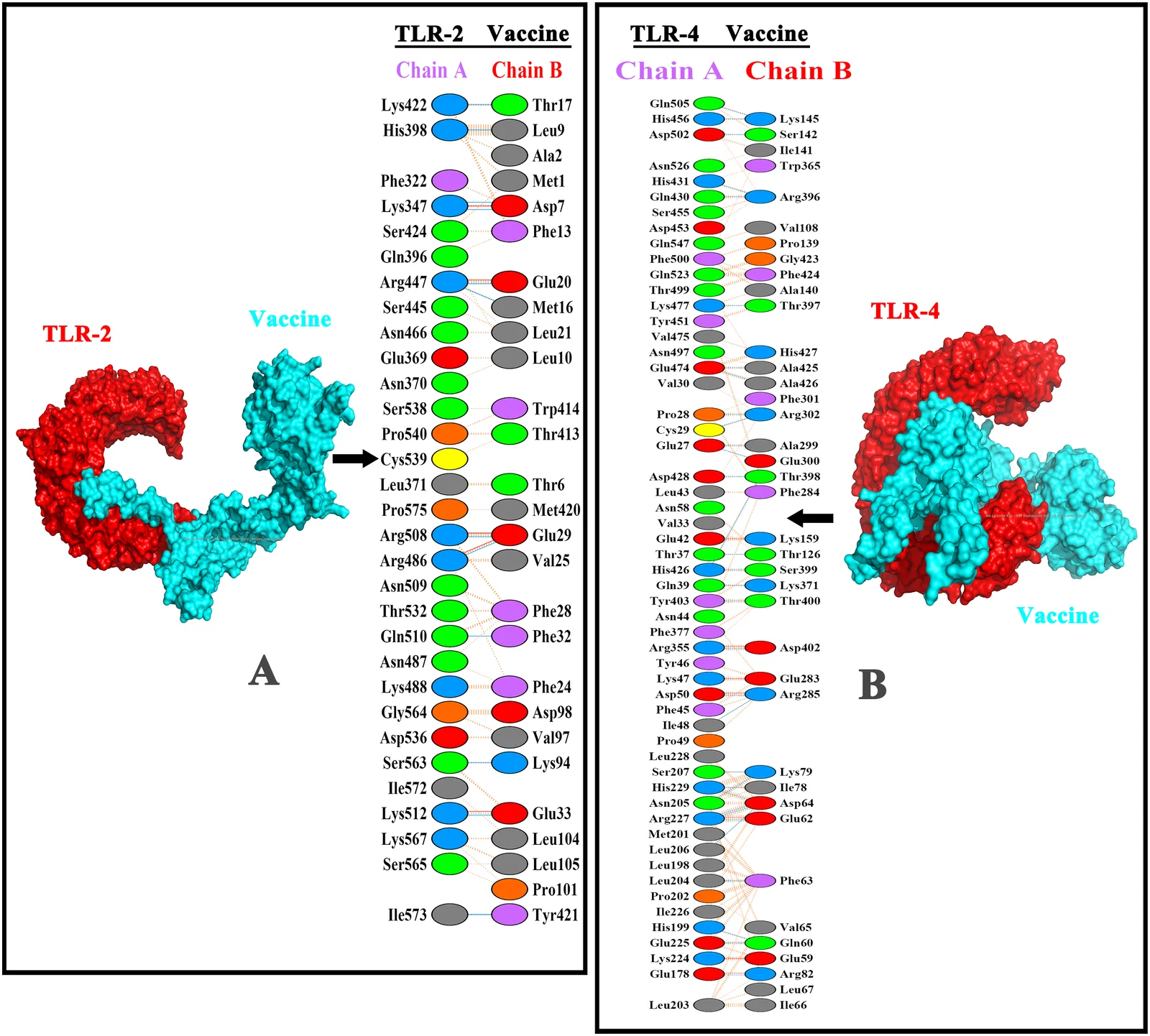
Docked complexes of Vaccine-TLR-2 (A) and Vaccine-TLR-4 (B).

### 3.12 Binding free energy (MM-GBSA)

The MM-GBSA was computed for both vaccine complexes over the HawkDock server. The server demonstrated binding free energy (−437.42 kcal/mol) for Vaccine-TLR-2, while VDW, GB, ELE, and SA were found (382.18 kcal/mol), (−839.96 kcal/mol), (−749.66 kcal/mol), and (−22 kcal/mol), respectively (Table 6). However, for vaccine- TLR-4 binding free energy determined to be (−217.08 kcal/mol), following that VDW, GB, ELE, and SA were found (−269.96 kcal/mol), (6855 kcal/mol), (−6768.36 kcal/mol) and (−33.77 kcal/mol) respectively (Table 6). Although individual MM-GBSA energy components (e.g., electrostatic and polar solvation terms) exhibit large magnitudes, these values largely compensate each other; therefore, the relative binding free energies are considered more reliable indicators for comparing complex stability.

**Table 6:** Binding free energy (MM-BGSA) analysis.

Complexes	VDW (kcal/mol)	ELE (kcal/mol)	GB (kcal/mol)	SA (kcal/mol)	TOTAL (kcal/mol)
Vaccine-TLR-2	382.18	−749.66	−839.96	−22	−437.42
Vaccine-TLR-4	−269.96	−6768.36	6855	−33.77	−217.08

### 3.13 Normal mode analysis

Three normal mode analyses (Vaccine-Apo, Vaccine-TLR-2, and Vaccine-TLR-2) were conducted using the IMOD server to assess structural integrity. The distance between residue indices in covariance maps indicated the relative positions of each amino acid in the protein sequence, with each point of the X and Y axes corresponding to a specific amino acid residue. Additionally, these maps used the hues red, white, and blue to illustrate the correlated, uncorrelated, and anti-correlated shifts between residue pairs of each complex, respectively. The sequence of the complexes exhibiting the highest correlation among residues was Vaccine-TLR-4 > Vaccine-TLR-2 > Vaccine-Apo, as indicated by the red colour (Fig 5A–5C). It is important to note that a stronger correlation in the covariance maps indicates a more effective complex. However, the matrix illustrated some marginal amounts of anti-correlated residues, as well as small amounts of uncorrelated residues in all the complexes, represented by blue and white colors across the matrix. Stronger correlated motions observed in the covariance maps suggest enhanced dynamic coupling within the complex, potentially contributing to its structural stability, rather than directly indicating biological effectiveness. The dynamics of a molecule were represented as a network of interconnected springs using an elastic network model to show a high degree of self-correlation among amino acid residues. Lighter patches, especially those off the diagonal, suggest weaker interactions, whereas darker hues along the diagonal indicate more robust elastic self-correlations. In this study, an elevated amount of dark grey zone on the diagonal line for all the complexes significantly indicates robust elastic correlations (Fig 5D–5F).

**Fig 5:**
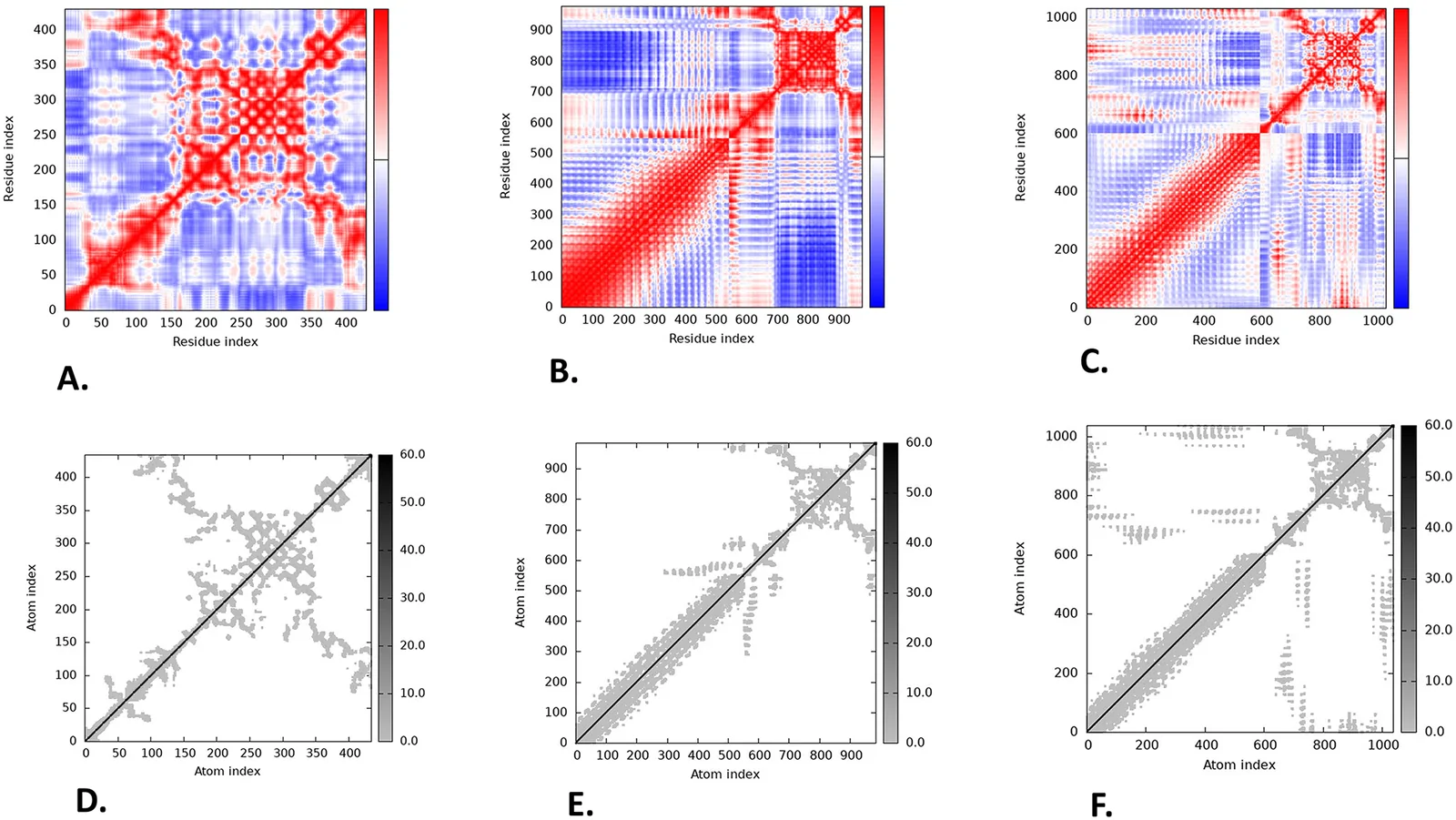
The normal mode analysis (covariance matrix) of Vaccine-Apo (A) Vaccine-TLR-2 (B) and Vaccine-TLR-4 (C) and Elastic network model Vaccine-Apo (D) Vaccine-TLR-2 (E) and Vaccine-TLR-4 (F).

To assess the extent of atomic motion or flexibility of the protein structure, B-factor graphs were generated. The B-factor was determined by utilizing the PDB structure in conjunction with normal mode analysis (NMA). The red line typically showed the anticipated flexibility of residues within the protein, with peaks indicating regions of higher predicted flexibility and valleys denoting more rigid regions. However, the order of the complexes with the most B-factors was Vaccine-TLR-2 > Vaccine-TLR-4 > Vaccine-Apo based on the peaks of red colors (S5A–S5C Fig).

Similarly, the deformability graphs depicted the extent of deformability at each position along the protein’s amino acid residue, represented in green, where “highest peaks” indicated regions of significant deformability. The sequence of the complexes exhibiting the highest deformability aligns with the sequence of the complexes characterized by their B factors. Nevertheless, the order of the complexes with the greatest deformability was Vaccine-TLR-2 > Vaccine-TLR-4 > Vaccine-Apo (S5D–S5F Fig).

In the eigenvalue graphs, the X-axis represented the index of the normal mode (ranging from mode 2 to mode 20), with these modes representing distinct deformation or vibration patterns of a protein, each associated with a unique frequency or motion pattern. The Y-axis illustrated the eigenvalue, indicating the impact of individual deformation movements on the overall protein motion, specifically the ratio of each mode’s eigenvalue to that of the first mode. The computed eigenvalues of Vaccine-Apo, Vaccine-TLR-2 and Vaccine-TLR-4 complexes were 1.435222e-06, 8.006343−07 and 9.627305−06, respectively (S6A–S6C Fig).

### 3.14 Molecular dynamics simulation

#### 3.14.1 Root mean square (RMSD) analysis.

The structural integrity of the apo and the Vaccine–TLR-4 complex was assessed using RMSD analysis during a 100 ns molecular dynamics simulation (Fig 6). The Vaccine–TLR-4 provided low RMSD values, in a range of 0.4 to 1.0 nm, representing the stability (Fig 6A). Conversely, the apo provided an uprising RMSD values, varying between 0.6 and 1.8 nm, signifying probable structural flexibility (Fig 6B).

**Fig 6:**
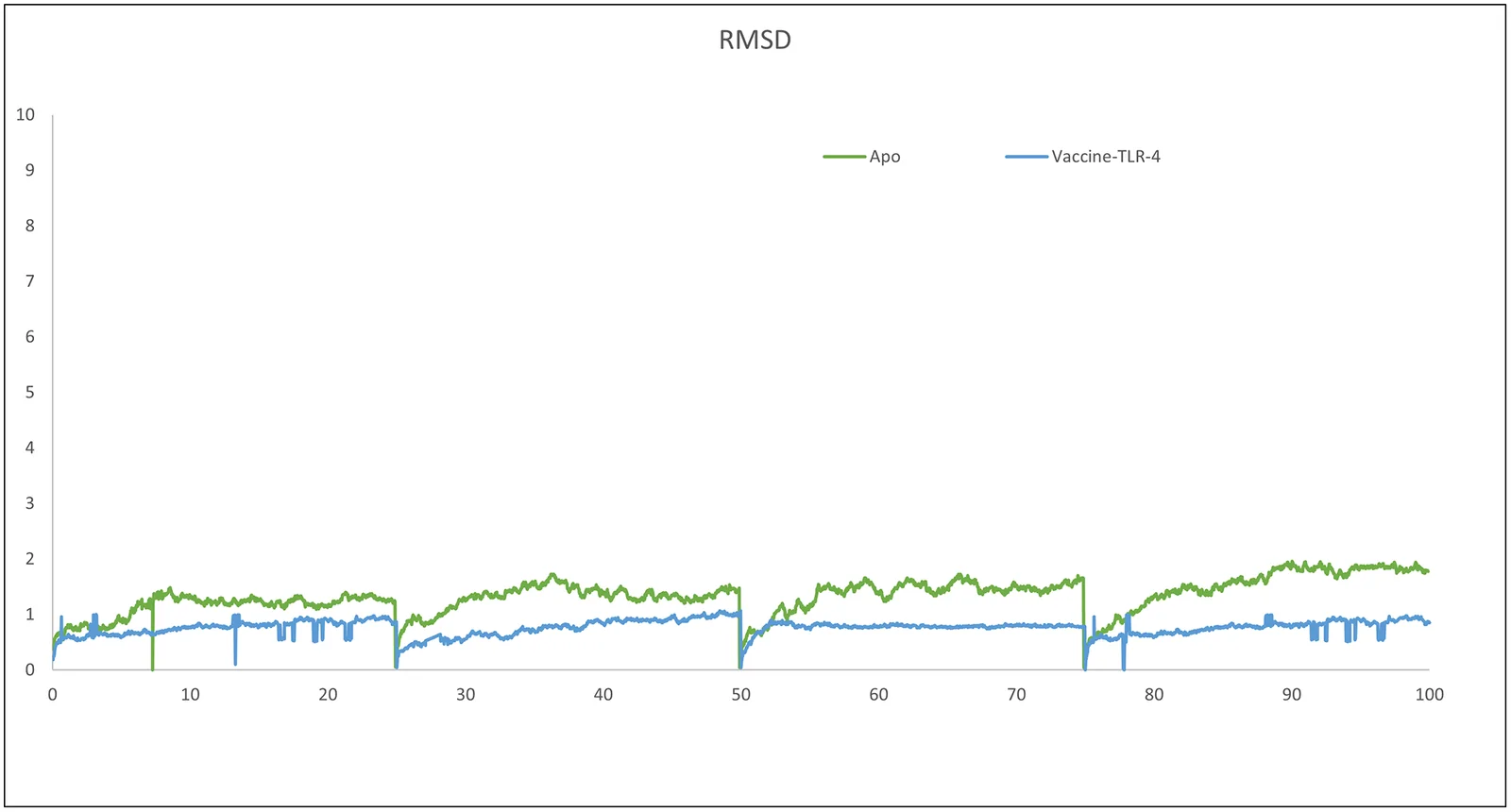
The RMSD analyses of the Apo (A) and Vaccine-TLR-4 (B).

#### 3.14.2 Root mean fluctuation (RMSF) analysis.

RMSF analysis was performed to assess the residue-level compactness of apo and the Vaccine–TLR-4 (Fig 7). The apo provided constant minimal fluctuations over the maximum residues, providing a probable well configuration (Fig 7A). The Vaccine-TLR-2 exhibited a nearly same fluctuating pattern, with slightly elevated values in certain residues and a significant increase in the central area, resulting in localized conformational alterations induced by ligand binding (Fig 7B).

**Fig 7:**
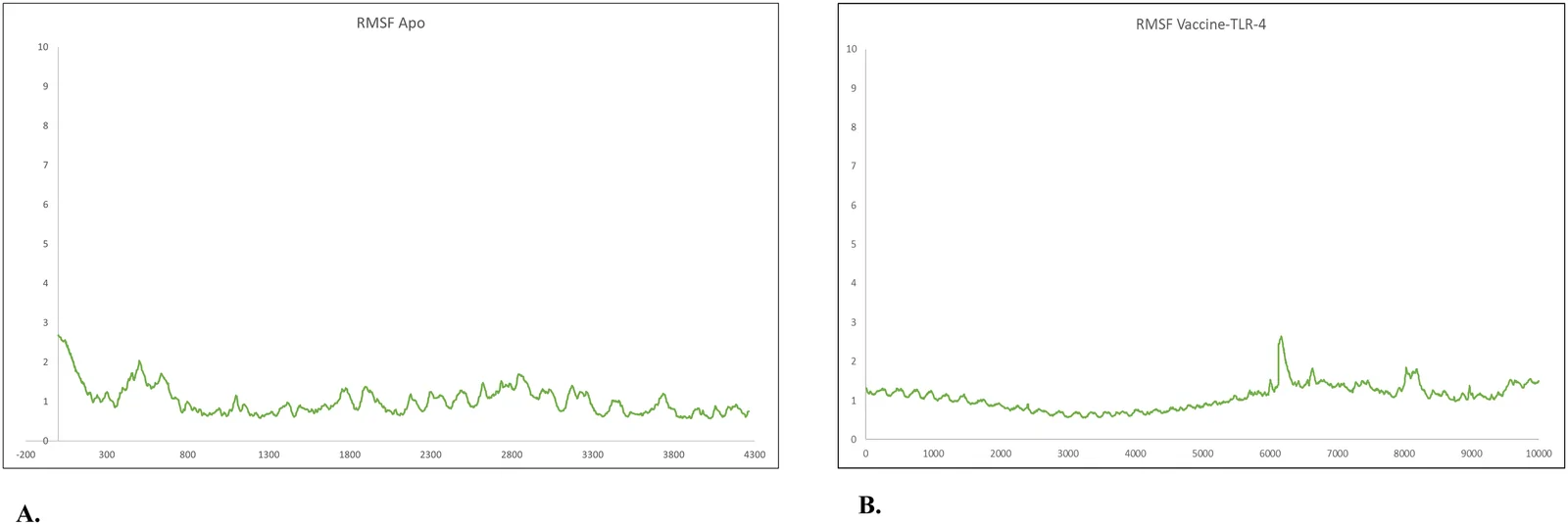
The RMSF analyses of the Apo (A) and Vaccine-TLR-4 (B).

#### 3.14.3 Solvent accessible surface area (SASA) analysis.

The level of the exposure of solvent for both apo and the Vaccine–TLR-4 was performed for checking the accessibility of the models (Fig 8A). The Vaccine–TLR-4 complex consistently exhibited higher SASA values than the apo across the entire simulated period. Both systems demonstrated minimal fluctuations without any abrupt or extended changes, indicating steady solvent exposure during the simulation. Notwithstanding the detection of slight temporary variations at different times, the SASA profiles demonstrated a relatively stable prediction.

**Fig 8:**
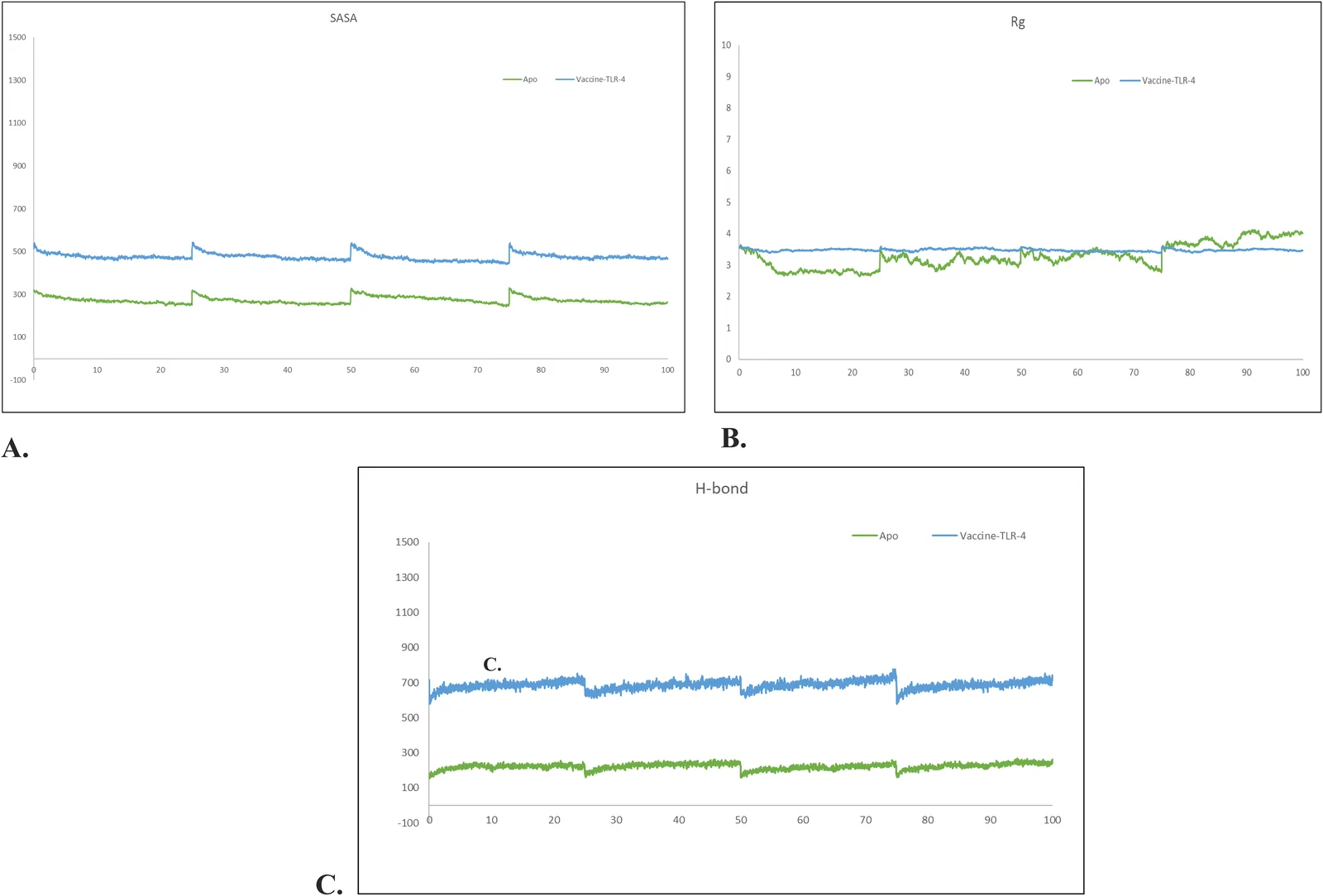
The SASA (A), Rg (B) and H bond (C) analyses of the Apo and Vaccine-TLR-4.

#### 3.14.4 Radius of gyration (Rg) analysis.

The compactness of apo and the Vaccine–TLR-4 complex was evaluated by observing the radius of gyration (Rg) throughout the 100 ns simulation (Fig 8B). The vaccine-associated complex exhibited a nearly stable Rg during the simulation, displaying only slight variations from its mean value. Conversely, the apo exhibited somewhat larger variations and a progressive rise in Rg over the last phases of the simulation, suggesting a minor decrease in structural density. The comparatively constant Rg profile of the Vaccine–TLR-4 complex indicates that the binding of the vaccine aids in maintaining the probable compact structural integrity of the receptor throughout the molecular dynamics simulation.

#### 3.14.5 Hydrogen bond analysis.

The robustness of intermolecular connections between the Vaccine and TLR-4 was additionally assessed by observing the quantity of hydrogen bonds established throughout the 100 ns simulation (Fig 8C). The Vaccine–TLR-4 complex constantly exhibited a significantly greater quantity of hydrogen bonds (500–700) compared to the apo receptor (150) during the simulation. Without any extended disruption of interactions, the hydrogen bond configuration demonstrated considerable stability, despite the observation of slight variations.

### 3.15 *In-silico* cloning and codon optimization

The *In-silico* cloning was assessed to incorporate the vaccine into the *E. coli* expression system. Subsequently, the codon of the vaccine was optimized within the *E. coli* K12 expression system utilizing the JCat server. This server demonstrated GC content (43.82) and CAI (1), both within standard ranges. Nonetheless, the values range from 0 to 1, which indicates successful expression. As the CAI score rises, the chance of gene expression increases equally. Following the optimization of the Condon sequence, the vaccine sequence was integrated into the pET 28(+) vector at the Eco53kI and EcoRv restriction sites with SnapGene Software (Fig 9).

**Fig 9:**
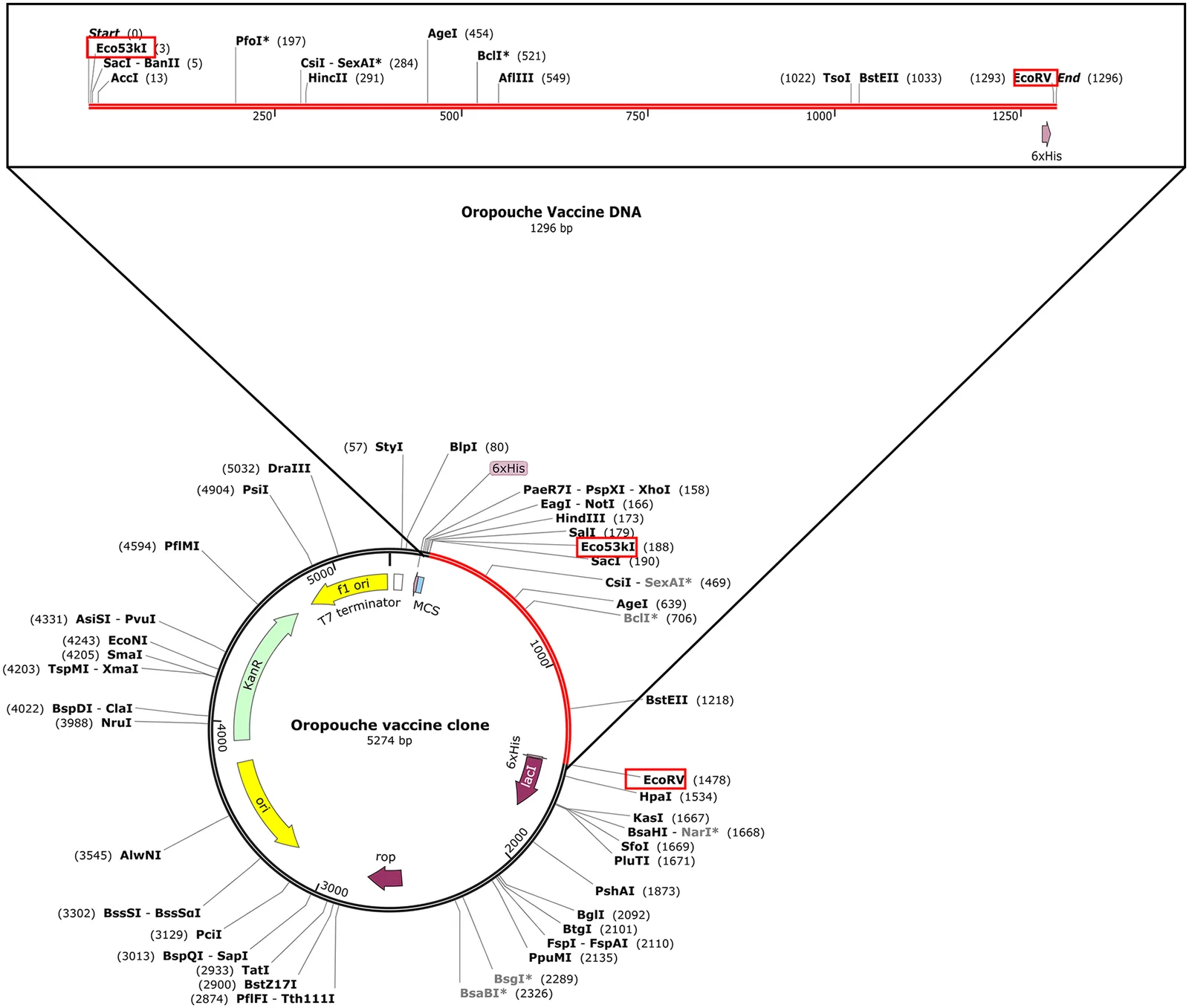
Codon optimization into the pET28a (+) vector by SnapGene software, where red boxes illustrate restriction sites (Eco53kI) and (EcoRV).

### 3.16 Immune response simulation

The vaccine exhibited a substantial immunological response in a computational immune simulation approach. A substantial increase in memory B-cell and total B-cell counts was observed. This is consistent with a year-long elevation following three rounds of vaccination (Figs 10A and S7). T cell responses such as TC, TR, and TH increased notably during the vaccination regimen. After 60 days, improved active and memory cell concentration were noticed (Figs 10C and S7). Furthermore, Active TR and TC expression levels notably increased after a year of the vaccination period (Figs 10E and S7B). Moreover, the post-vaccine period also showcased significant innate immune responses, for example, DC (Fig 10D), NK (Fig 10B), and MA (S7D Fig) responses. These results predict that this vaccine can probably induce various immune cells. There was also a cytokine response, although it could not be seen 2 months post-vaccination (Fig 10F).

**Fig 10:**
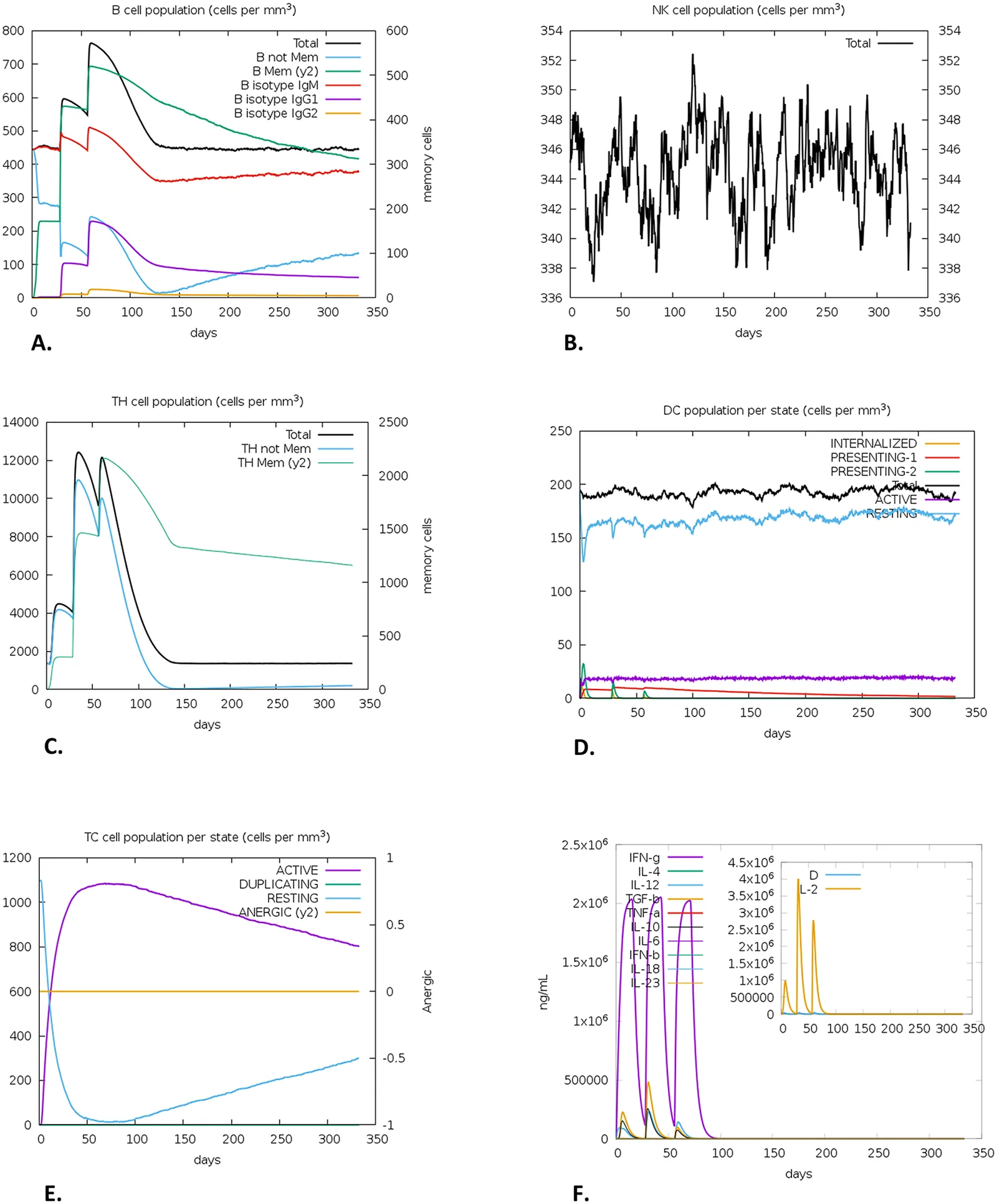
Immune simulation of OROV vaccine with B-cell population (A), NK-cell population (B), TH-cell population (C), DC population per state (D), TC-cell population per state (E) and cytokines (F).

### 3.17 mRNA structure validation

The mRNA secondary structure of the vaccine was illustrated through the RNAfold server (S8 Fig), with minimum free energy (MFE) scores −293.50 kcal/mol (optimal secondary structure) and −202.40 kcal/mol (centroid secondary structure). The frequency of MFE in the ensemble and the energy of thermodynamics were predicted to be 0.00% and −316.70 kcal/mol, respectively.

## 4. Discussion

A vaccine is considered the most vital lifesaving component after water. However, it is challenging to manufacture a suitable vaccine candidate [64]. In the post-genomic era, several strategies have been used to identify the appropriate antigen. Among all techniques, the immunoinformatics and RV approach have demonstrated its great potential during the COVID-19 period. Similarly, this approach is also implemented to identify vaccine candidates for other diseases such as the Zika virus, influenza, and chikungunya [65]. Therefore, in this study, the RV and immunoinformatics were employed to determine an ideal vaccine candidate for OROV. Despite the increasing application of these approaches for vaccine development against emerging arboviruses, only a limited number of studies have specifically focused on multiepitope subunit-based vaccine design against Oropouche virus (OROV). Among these, recent studies have utilized an *in-silico* approach to design multi-epitope vaccines. Naveed M., et al., (2026) primarily focused on the envelope polyprotein. They employed an advanced immunoinformatics workflow incorporating molecular docking and molecular dynamics (MD) simulations, reporting a stable Vaccine–TLR-4 complex with an RMSD of ~0.40 nm and a binding energy of −1067.1 kJ/mol. However, this study was conducted on a single antigenic protein and, Das T., et al., (2025) expanded the antigenic scope by selecting epitopes from multiple proteins and designing a vaccine construct incorporating CTL, HTL, and B-cell epitopes along with regulatory elements, however, it lacks rigorous dynamic validation and detailed structural refinement [66,67].

This study focused on developing a multi-epitope subunit vaccine against OROV, which includes the nucleocapsid (N) protein, G1 and G2 glycoproteins, and B-cell epitopes from putative MHC-I, MHC-II, and B-cell proteins. Neutralizing antibodies must target the surface-exposed glycoproteins G1 and G2, which are crucial in host cell adhesion and membrane fusion. On the other hand, T-cell-mediated immunity relies heavily on the nucleocapsid protein (N), which is extremely conserved. Prior research did not integrate these structurally and functionally similar antigens concurrently; instead, it focused on either a single viral protein or a collection of viral proteins. As a result, our vaccine design provides greater immunological coverage by enhancing both humoral and cellular immune responses through the inclusion of G1, G2, and N proteins.

While all epitopes are important, CTL epitopes activate CD8 + T-cell responses by MHC-I presentation, allowing the elimination of virus-infected cells, and HTL epitopes stimulate CD4 + T-cell responses through MHC-II pathways, contributing to cellular and antibody-mediated immunity. Here, T-cell responses were the focus of Naveed M. et al. (2026), whereas CTL, HTL, and B-cell epitopes were included by Das T. et al. (2025). But our research makes sure that all three types of epitopes were included [66,67]. Demographic coverage analysis provided additional validation, showing that our vaccine design had a predicted HLA coverage of 100%, which was higher than the 94.07% coverage reported in the study by Naveed M. et al. (2026) and the region-specific values published in the study by Das T. et al. (2025). These results provide that our vaccine can be used by a wide range of people [66,67]. Nonetheless, the demographic coverage was entirely based on computational predictions rather than a conclusive assertion. It necessitates empirical confirmation. Additional evidence of the vaccine’s durability and appropriateness was provided by its biophysical characteristics. The instability index of our construct was 18.29, which was much lower than the previously reported values of 39.82 and 30.23, suggesting that it was more stable. The aliphatic index of 78.66 indicated good thermostability, and the GRAVY score of −0.432 verified favorable solubility. Taken together, these metrics show that the engineered vaccine had better biophysical properties that help with expression efficiency and in vivo stability. Ramachandran plot analysis revealed 91.86% residues in the most favored regions, 7.21% in allowed regions, and only 0.93% in generously allowed regions, indicating a well-folded and stereochemically reliable structure compared to Naveed M., et al., (2026) and Das T., et al., (2025) reported values (~82–88% in favored regions) [66,67]. Additionally, the ERRAT score of 71.85 exceeds the acceptable threshold (>50), indicating reliable non-bonded interactions, while the Z-score of −1.81 further supports the structural validity of the model The interaction between the vaccine and immune receptors was evaluated through molecular docking analysis with TLRs. Naveed M., et al., (2026) and Das T., et al., (2025) reported docking energies in the range of −646.6 to −771.8 kJ/mol (TLR-2) and −102.4 to −113.2 kJ/mol (TLR-4) [66,67]. Conversely, our vaccine construct showed predicted good interactions, with docking scores of −1126.8 kJ/mol (Vaccine-TLR-2) and −1239.4 kJ/mol (Vaccine-TLR-4). By combining features such as multi-protein targeting (G1, G2, and N), rigorous epitope selection, high population coverage, superior biophysical stability (instability index 18.29), robust structural validation (91.86 percent favored residues), and dramatically improved receptor binding affinity, the subunit vaccine developed in this study not only builds on but also overcomes the shortcomings of earlier methods. Consequently, our research offers a more all-encompassing and strategically optimal framework for vaccine design, which has a probable better chance of eliciting a robust, consistent, and efficient immune response against OROV.

The theoretical isoelectric point (pI) value was 9.95, indicating that it was a basic protein [68]. The aliphatic index of 78.66 suggested that the vaccine exhibited considerable thermal stability [68]. The significant excellence of this vaccine was indicated by an instability index of 18.29 (<40, indicating stability), which is lower than the threshold. The binding free energy of Vaccine-TLR-2 (−450.49 kcal/mol) and Vaccine-TLR-4 (−217.08 kcal/mol) indicates a robust binding affinity. For Vaccine-TLR-2, the values for Van der Waals (VDW), generalized Born (GB), electrostatic (ELE), and solvent-accessible surface area (SA) were determined to be 382.18 kcal/mol, 839.96 kcal/mol, −749.66 kcal/mol, and −22 kcal/mol, respectively. Conversely, for the Vaccine-TLR-4 VDW, the binding energies were determined as follows: GB at −269.96 kcal/mol, ELE at −6855 kcal/mol, SA at −6768.36 kcal/mol, and the total at −33.77 kcal/mol. The values indicate robust molecular interaction between the vaccine and receptor. The MM-GBSA study was carried out with the HawkDock server, which uses the AMBER force field to calculate the binding free energy of protein-protein complexes. The electrostatic and polar solvation energy levels were rather high but had opposing signs. This is a common feature of MM-GBSA computations, as the energy required to remove surrounding water molecules during binding more than offsets beneficial electrostatic interactions. As a result, these distinct energy terms should not be interpreted independently. The overall binding free energy (ΔG), which includes all energy contributions, is regarded as the most reliable indication of binding affinity. This study’s negative ΔG values indicate that vaccine-receptor complexes are both stable and energy-efficient. However, MM-PBSA/MM-GBSA calculations that are conducted using representative frames extracted from an equilibrated molecular dynamics trajectory provide a more comprehensive estimation of binding free energy than docking scores alone, as they account for conformational changes that occur during the simulation. So, this is a limitation not to perform the trajectory-based analysis but in the near future, it will be conducted. The IMOD normal mode analysis was employed to assess the predicted structural integrity of the vaccine along with complexes. The eigenvalues of three vaccine-receptor complexes were as follows: Vaccine-Apo (1.435222e-06), Vaccine-TLR-2 (8.006343e-07), and Vaccine-TLR-4 (9.627305e-06). The low eigenvalues signified low-frequency vibrations, indicating that the complex is probably structurally stable and demonstrates probable restricted internal flexibility in those modes. The Vaccine-TLR-2 complex had some of the lowest values, indicating a stiff, tightly bound interaction between the Vaccine and the receptor. Nonetheless, the maximum value for the Vaccine-TLR-4 complex indicates probable greater flexibility in that specific vibrational mode, albeit within a stable range. Subsequently, the molecular dynamics simulation analysis indicate that the vaccine establishes a probable stable and dynamically advantageous complex with TLR-4 over the course of the 100 ns simulation. The RMSD analysis demonstrated that the Vaccine–TLR-4 complex exhibited persistently reduced structural deviations compared to the apo throughout the simulation, signifying improved probable conformational stability upon ligand interaction. Although brief reductions in RMSD were seen at about 25, 50, and 75 ns, these changes were soon recovered to their equilibrium levels and hence presumably represent trajectory processing or periodic structural realignment rather than genuine conformational instability. The whole RMSD profile indicates that the receptor–vaccine combination swiftly reached equilibrium and maintained structural stability across the simulation duration. The flexibility at the residue level was additionally evaluated by RMSF analysis. The receptor associated with the immunisation demonstrated localised enhancements in variability compared to the apo protein, especially in some loop areas, but most residues displayed minimal mobility. Localised flexibility is often linked to adaptive conformational changes that enhance ligand binding and improve intermolecular interactions while maintaining the overall structural integrity of the receptor. The lack of significant variations suggests that vaccine binding causes only minor structural changes while maintaining the overall stability of TLR-4. The SASA revealed a consistent probable solvent accessibility during the simulation for both systems. The Vaccine–TLR-4 complex exhibited a somewhat greater solvent-accessible surface area compared to the apo, although no significant alterations or gradual increases in solvent exposure were detected over time. This indicates that complex formation does not lead to much unfolding or structural enlargement and that the receptor preserves a stable tertiary configuration throughout the simulation. In conjunction with the SASA results, the radius of gyration (Rg) assessment revealed that the vaccine-bound complex maintained a relatively stable degree of structural compactness during the simulation. Conversely, the apo had relatively larger variations and a minor inclination towards elevated Rg values in the latter phases of the trajectory, indicating enhanced inherent conformational flexibility. The consistent Rg profile of the vaccine-bound system suggests that ligand attachment aids in preserving the compact structural arrangement of TLR-4. The analysis of hydrogen bonds further corroborated the stability of the receptor-vaccine connection. The complex constantly exhibited a significant quantity of hydrogen bonds during the simulation, although slight temporal variations. Enduring hydrogen bonding is a crucial factor in stable protein-ligand interactions, enhancing conformational stability and minimising the chances of complex breakup in fluctuating environments. The expression of a recombinant vaccine in an *E. coli* vector was assessed by codon optimization. A GC percentage of 43.82% and a CAI value of 1 indicated a substantial expression level within the vector. For a recombinant subunit vaccine, computer analysis alone cannot be used to identify reliably the appropriate dose and delivery formulation. Based on standard procedures for recombinant protein vaccines, the purified vaccine design might be first assessed using intramuscular or subcutaneous administration with an appropriate authorized adjuvant or delivery system. Possible formulations include aluminium-based adjuvants, oil-in-water emulsions or biodegradable nanoparticle-based delivery methods. These formulations may improve the stability of the antigen, its uptake by antigen-presenting cells, and the generation of both humoral and cellular immune responses [69]. The presence of memory B cells and T cells in the vaccine indicated activation of both adaptive and innate immune responses. Furthermore, the immunoglobulin data (IgM and IgG) suggest a hormonal immunological response. Finally, the stability of the RNA structure was predicted to be stable but it requires experimental validation. The RNAfold-derived MFE values were used as a preliminary *in-silico* indicator of structural stability rather than definitive proof of biological stability. The study examines the suitability of the developed OROV subunit vaccine and the effectiveness of robust cellular and humoral immune responses to the epitopes of polyprotein (G1 and G2) and nucleocapsid protein. The modelled three-dimensional conformation demonstrated satisfactory stereochemical integrity; however, the suggested vaccine is a synthetically designed fusion protein that includes various epitopes, linkers, an adjuvant, and supplementary functional sequences. Thus, the predicted structure should to be considered as a computational prediction rather than an absolute representation of the natural protein configuration. Similarly, molecular docking results depend on how accurate the predicted structure is, and they don’t take into account protein folding, conformational flexibility, or the changing ways molecules interact in living things. As a result, the structural modelling and docking studies give us important first clues about how the vaccine construct might work, but their results need to be confirmed through structure characterization experiments and biological validation. Despite that, this study mostly looked at computer predictive analytics, which has some problems. These problems include not being able to predict long-term immunity, making wrong structure predictions, using theoretical immune modelling that isn’t very accurate, not knowing how adjuvants will interact with different epitopes, proteins that aren’t stable, proteins that can cause allergies or toxicity by accident, and more. So, to make sure that this research is correct, it needs to go through both preclinical and clinical trials, as well as being closely watched for a long time by regulatory bodies like the Food and Drug Administration and the Centers for Disease Control and Prevention.

## 5. Conclusion

Reverse vaccinology has demonstrated efficacy across all areas, including the development of the SARS-CoV-2 vaccine. This study aims to develop a multiepitope subunit vaccine candidate against OROV by targeting proteins responsible for infection. The study’s results suggest that the vaccine could probably elicit a robust immune response without causing allergic reactions or toxicity. Additionally, the selected epitopes showed probable high conservation and probable substantial population coverage across diverse regions worldwide. Molecular docking scores indicated probable high-affinity interactions with TLR-2 and TLR-4 receptors, which were further validated by MM-GBSA and dynamics simulation analysis. The vaccine also showed potent probable cloning ability when expressed in a bacterial vector, and simulations of immunological responses suggested it could probable stimulate both cell-mediated and antibody-mediated immune responses. Overall, the favorable computational findings improve the proposed vaccine candidate’s translational potential. The favorable physicochemical features, together with successful probable codon optimization and *in-silico* cloning, suggest the viability of recombinant production for further experimental studies. Although these findings need to be validated *in-vitro* and *in-vivo*, they provide compelling evidence for developing this multi-epitope subunit vaccine as a possible preventive candidate against Oropouche virus.

## Supporting information

S1 Fig Demographic coverage analysis for MHC-I and MHC-II epitopes for Oropouche vaccine mapping. (PNG)

S2 Fig The secondary structure of the OROV vaccine by PSIPRED. (PNG)

S3 Fig The disulfide engineering of the OROV vaccine with (A) the raw model and the (B) mutant model with disulfide bonds in cyan color. (TIF)

S4 Fig Structural B-cell epitopes (A-D), where the yellow section predicted B-cell epitopes, and the grey sticks indicated the vaccine. (TIF)

S5 Fig The normal mode analysis (B-factor) of Vaccine-Apo (A), Vaccine-TLR-2 (B) and Vaccine-TLR-4 (C) and deformability graph, Vaccine-Apo (D), Vaccine-TLR-2 (E) and Vaccine-TLR-4 (F). (PNG)

S6 Fig The normal mode analysis (eigenvalue) Vaccine-Apo of (A) Vaccine-TLR-2, (B) and Vaccine-TLR-4 (C). (PNG)

S7 Fig Immune simulation of Oropouche vaccine with B-cell population per state (A), TR-cell population per state (B), TH-cell population per state (C), MA population per state (D), TC-cell population (E) and antigen/antibody titers (F). (PNG)

S8 Fig mRNA structure of vaccine by RNA fold server, Base pair probabilities with minimum free energy (A) and centroid (B), positional entropy of vaccine with minimum free energy (C) and centroid (D). (PNG)

S1 Table The list of HLA MHC-1 and HLA MHC-2 alleles. (DOCX)

S2 Table The two-dimensional structure findings of identified vaccine. (DOCX)

S3 Table The amino acid residues, Chi3 angle, energy and B factor values of the disulfide engineering of the OROV vaccine model. (DOCX)

S4 Table The structural B-cell epitopes residues of the vaccine. (DOCX)

S1 File More files for reproducibility. (RAR)
