# Supplementary material for: An immunoinformatics-driven multiepitope subunit vaccine targeting Oropouche virus: Molecular docking, immune and dynamics simulation approaches
Source: PLoS One. 2026 Oct 9;21(10):e0360186. doi: 10.1371/journal.pone.0360186 (PMC13652280; doi:10.1371/journal.pone.0360186)
Supplement: Supplementary file 2 [file pone.0360186.s010.docx]

**Supplementary Table 2.** The two-dimensional structure findings of identified vaccine.

| Properties | GOR4 | SOPMA |
| --- | --- | --- |
| Alpha helix (Hh) | 213 (49.31%) | 163 (37.73%) |
| 3_10_helix (Gg) | 0.00% | 0.00% |
| Pi helix (Ii) | 0.00% | 0.00% |
| Beta bridge (Bb) | 0.00% | 0.00% |
| Extended strand (Ee) | 62 (14.35%) | 70 (16.20%) |
| Beta turn (Tt) | 0.00% | 14 (3.66%) |
| Bend region (Ss) | 0.00% | 0.00% |
| Random coil (Cc) | 157 (36.34%) | 177 (40.97%) |
