# Supplementary material for: An immunoinformatics-driven multiepitope subunit vaccine targeting Oropouche virus: Molecular docking, immune and dynamics simulation approaches
Source: PLoS One. 2026 Oct 9;21(10):e0360186. doi: 10.1371/journal.pone.0360186 (PMC13652280; doi:10.1371/journal.pone.0360186)
Supplement: Supplementary file 3 [file pone.0360186.s011.docx]

**Supplementary Table 3.** The amino acid residues, Chi3 angle, energy and B factor values of the disulfide engineering of the OROV vaccine model.

| **Res1 Chain** | **Res1 Seq #** | **Res1 AA** | **Res2 Chain** | **Res2 Seq #** | **Res2 AA** | **Chi3** | **Energy** | **Sum B-Factors** |
| --- | --- | --- | --- | --- | --- | --- | --- | --- |
| A | 5 | SER | A | 8 | GLU | 110.72 | 4.78 | 17.77 |
| A | 36 | ALA | A | 39 | PRO | 86.42 | 4.39 | 10.79 |
| A | 39 | PRO | A | 41 | ALA | -109.61 | 2.38 | 8.79 |
| A | 44 | ALA | A | 69 | ALA | -106.09 | 2.26 | 8.56 |
| A | 76 | GLY | A | 81 | VAL | -68.29 | 3.19 | 12.82 |
| A | 103 | PRO | A | 420 | MET | 106.57 | 2.74 | 17.45 |
| A | 153 | GLY | A | 359 | THR | -111.28 | 6.22 | 9.29 |
| A | 178 | LYS | A | 182 | LYS | 108.9 | 5.31 | 9.5 |
| A | 185 | VAL | A | 194 | LYS | 114.17 | 4.02 | 9.24 |
| A | 185 | VAL | A | 235 | GLN | 116.16 | 2.76 | 9.2 |
| A | 187 | PHE | A | 190 | GLN | 81.67 | 7.28 | 9.31 |
| A | 201 | LEU | A | 204 | VAL | 67.11 | 7.9 | 18.35 |
| A | 225 | LYS | A | 341 | GLY | -74.22 | 3.05 | 11.67 |
| A | 243 | LYS | A | 315 | LYS | 99.74 | 4.49 | 9.68 |
| A | 252 | SER | A | 305 | VAL | 118.95 | 3.68 | 13.5 |
| A | 254 | THR | A | 344 | VAL | -74.42 | 8.11 | 10.91 |
| A | 258 | LYS | A | 339 | ILE | -115.46 | 4.16 | 9.78 |
| A | 263 | THR | A | 340 | SER | 98.3 | 1.49 | 10.82 |
| A | 264 | VAL | A | 330 | SER | -100.72 | 3.86 | 9.1 |
| A | 266 | SER | A | 276 | THR | -92.52 | 3.3 | 9.52 |
| A | 266 | SER | A | 319 | GLU | -103.81 | 7.25 | 9.12 |
| A | 268 | ASN | A | 317 | ASN | 109.37 | 3.03 | 9.52 |
| A | 270 | PRO | A | 273 | GLU | 99.32 | 3.67 | 9.64 |
| A | 273 | GLU | A | 293 | ALA | 115.55 | 3.76 | 9.69 |
| A | 280 | LYS | A | 283 | GLU | 121.45 | 4.15 | 9.33 |
| A | 356 | ILE | A | 378 | SER | 79.66 | 5.78 | 9.05 |
| A | 366 | PHE | A | 393 | VAL | -116.08 | 4.12 | 9.45 |
| A | 366 | PHE | A | 395 | GLN | 98.71 | 3.02 | 9.44 |
| A | 367 | LYS | A | 390 | LYS | -90.38 | 4.18 | 9.32 |
| A | 369 | THR | A | 390 | LYS | 81.53 | 4.83 | 9.27 |
| A | 402 | ASP | A | 405 | ALA | 115.29 | 6.56 | 19.14 |
| A | 405 | ALA | A | 410 | LYS | 100.51 | 3.15 | 14.06 |
| A | 414 | TRP | A | 416 | ASP | -113.28 | 6.56 | 9.95 |
| A | 422 | LEU | A | 424 | PHE | -99.68 | 3.12 | 15.06 |
| A | 424 | PHE | A | 427 | HIS | 82.62 | 4.59 | 9.94 |
