# Supplementary material for: An immunoinformatics-driven multiepitope subunit vaccine targeting Oropouche virus: Molecular docking, immune and dynamics simulation approaches
Source: PLoS One. 2026 Oct 9;21(10):e0360186. doi: 10.1371/journal.pone.0360186 (PMC13652280; doi:10.1371/journal.pone.0360186)
Supplement: Supplementary file 4 [file pone.0360186.s012.docx]

**Supplementary Table 4.** The structural B-cell epitopes residues of the vaccine.

| Serial number | Residues | Number of residues | Scores |
| --- | --- | --- | --- |
| 1 | A:M1, A:A2, A:K3, A:L4, A:S5, A:T6, A:D7, A:E8, A:L9, A:L10, A:D11, A:A12, A:F13, A:K14, A:E15, A:M16, A:T17, A:L18, A:L19, A:E20, A:L21, A:S22, A:D23, A:F24, A:V25, A:K26, A:K27, A:F28, A:E29, A:E30, A:T31, A:F32, A:E33, A:V34, A:T35, A:A36, A:A37, A:A38, A:P39, A:V40, A:A41, A:V42, A:A43, A:A44, A:A45, A:G46, A:A47, A:A48, A:P49, A:A50, A:G51, A:A52, A:A53, A:V54, A:E55, A:A56, A:A57, A:E58, A:E59, A:Q60, A:S61, A:E62, A:F63, A:D64, A:V65, A:I66, A:L67, A:E68, A:A69, A:A70, A:G71, A:D72, A:K73, A:K74, A:I75, A:G76, A:V77, A:I78, A:K79, A:V80, A:V81, A:R82, A:I84, A:V85, A:S86, A:G87, A:L88, A:P103, A:L104, A:L105, A:E106, A:K107, A:V108, A:A109, A:T137, A:I138, A:P139, A:A140, A:I141, A:S142, A:G143, A:L144, A:K145, A:K146, A:G364, A:W365, A:F366, A:Q395, A:R396, A:T397, A:T398, A:S399, A:T400, A:F401, A:D402, A:A405, A:A406, A:Y407, A:K408, A:K410, A:G411, A:C412, A:T413, A:W414, A:N415, A:G417, A:D418, A:A419, A:M420, A:Y421, A:L422, A:G423, A:F424, A:A425, A:A426, A:H427, A:H428, A:H429, A:H430, A:H431, A:H432 | 141 | 0.727 |
| 2 | A:T177, A:K178, A:S179, A:Y180, A:K181, A:K182, A:V185, A:F186, A:F187, A:L188, A:N189, A:Q190, A:K191, A:K192, A:K193, A:K194, A:K195, A:T196, A:V197, A:V198, A:V199, A:P200, A:L201, A:A202, A:E203, A:V204, A:K205, A:K206, A:K207, A:L208, A:S209, A:A210, A:S211, A:F212, A:Q213, A:N214, A:T215, A:T217, A:N218, A:Q219, A:L220, A:P221, A:T222, A:K224, A:K225, A:K226, A:L227, A:S228, A:A229, A:S230, A:F231, A:Q232, A:N233, A:S257, A:K258, A:K259, A:K260, A:K261, A:A322, A:I323, A:T324, A:A325, A:I328, A:M329, A:S330, A:L331, A:K332, A:K333, A:K334, A:T335, A:I336, A:P337, A:A338, A:I339 | 74 | 0.654 |
| 3 | A:K351, A:K352, A:K353, A:F354, A:E355, A:I356, A:S378, A:K379, A:N380, A:K381, A:P382, A:A383, A:L384, A:E385, A:A386, A:T387 | 16 | 0.654 |
| 4 | A:K171, A:N237, A:Q238, A:L239, A:P240, A:K241, A:K242, A:K243, A:K244, A:D245, A:D246, A:I247, A:N268, A:K269, A:P270, A:A271, A:L272, A:E273, A:A274, A:L292, A:A293, A:E294, A:A310, A:E311, A:V312, A:K313, A:K314, A:C316, A:N317 | 29 | 0.519 |
