# Supplementary material for: An immunoinformatics-driven multiepitope subunit vaccine targeting Oropouche virus: Molecular docking, immune and dynamics simulation approaches
Source: PLoS One. 2026 Oct 9;21(10):e0360186. doi: 10.1371/journal.pone.0360186 (PMC13652280; doi:10.1371/journal.pone.0360186)
Supplement: Supplementary file 5 [file pone.0360186.s015.docx]

Dear respected editor and reviewers, we acknowledge your suggestions and we have addressed the suggestions in the revised manuscript. Your suggestions will help us improve the manuscript.

**Journal Requirements**

1. Please ensure that your manuscript meets PLOS ONE's style requirements, including those for file naming. The PLOS ONE style templates can be found at

<https://journals.plos.org/plosone/s/file?id=wjVg/PLOSOne_formatting_sample_main_body.pdf> and

[https://journals.plos.org/plosone/s/file?id=ba62/PLOSOne_formatting_sample_title_authors_affiliations.pdf](https://track.editorialmanager.com/CL0/https:%2F%2Fjournals.plos.org%2Fplosone%2Fs%2Ffile%3Fid=ba62%2FPLOSOne_formatting_sample_title_authors_affiliations.pdf/1/010f019d89b36dc4-3de8e266-350c-4c34-9169-2f7ab0ca2458-000000/AUWtf4nLn0vrAIw-qOazpNb1xOkHWMpzdzDOTJPJvEs=258)

**Response:** The manuscript has been checked for the requirements of PLOS ONE's style and it was found that it meets the requirements.

2. Please note that PLOS One has specific guidelines on code sharing for submissions in which author-generated code underpins the findings in the manuscript. In these cases, we expect all author-generated code to be made available without restrictions upon publication of the work. Please review our guidelines at <https://journals.plos.org/plosone/s/materials-and-software-sharing#loc-sharing-code> and ensure that your code is shared in a way that follows best practice and facilitates reproducibility and reuse.

**Response:** We have reviewed the PLOS ONE code-sharing guidelines and will ensure that all author-generated code underpinning the findings of this manuscript is made publicly available without restrictions upon publication.

3. When completing the data availability statement of the submission form, you indicated that you will make your data available on acceptance. We strongly recommend all authors decide on a data sharing plan before acceptance, as the process can be lengthy and hold up publication timelines. Please note that, though access restrictions are acceptable now, your entire data will need to be made freely accessible if your manuscript is accepted for publication. This policy applies to all data except where public deposition would breach compliance with the protocol approved by your research ethics board. If you are unable to adhere to our open data policy, please kindly revise your statement to explain your reasoning and we will seek the editor's input on an exemption. Please be assured that, once you have provided your new statement, the assessment of your exemption will not hold up the peer review process.

**Response:** Thank you for your clarification regarding the data availability policy. The data is already available within the manuscript and the supplementary information.

4. We note you have included a table to which you do not refer in the text of your manuscript. Please ensure that you refer to Table 2 in your text; if accepted, production will need this reference to link the reader to the Table.

**Response:** The Table 2 has been referred to the revised manuscript

5. Please include captions for your Supporting Information files at the end of your manuscript, and update any in-text citations to match accordingly. Please see our Supporting Information guidelines for more information: [http://journals.plos.org/plosone/s/supporting-information](http://track.editorialmanager.com/CL0/http:%2F%2Fjournals.plos.org%2Fplosone%2Fs%2Fsupporting-information/1/010f019d89b36dc4-3de8e266-350c-4c34-9169-2f7ab0ca2458-000000/j7DMCFotrOEaRG5u0tL1ltjy9AKIZxRnvsrpIs7Ym9Q=258).

**Response:** The captions for the Supporting Information files have been added at the end of the revised manuscript.

6. If the reviewer comments include a recommendation to cite specific previously published works, please review and evaluate these publications to determine whether they are relevant and should be cited. There is no requirement to cite these works unless the editor has indicated otherwise.

**Response:** Thank you for your clarification. No author has told us to cite any published articles.

Reviewer's Responses to Questions

**Reviewer #1: Questions**

1. The manuscript describes the design of an mRNA vaccine; however, several aspects of the workflow appear more consistent with recombinant protein expression strategies. Examples include the addition of a His-tag (lines 159–160), the use of a bacterial adjuvant (50S ribosomal protein of M. tuberculosis, lines 156–157), codon optimization for E. coli (section 3.14, lines 408–413), and cloning into the pET-28a(+) vector (lines 414–415). These elements are typically associated with bacterial protein expression rather than mRNA vaccine design. Clarification of the intended vaccine platform would therefore be helpful.

**Response:** Thank you for your valuable observation. We agree that several components of the workflow, including the inclusion of the His-tag, the use of the 50S ribosomal protein adjuvant, codon optimization for *E. coli*, and cloning into the pET-28a(+) vector, are commonly associated with recombinant protein expression systems rather than conventional mRNA vaccine platforms. However, in the methodology and image of the vaccine construction of the revised manuscript, the other essential mRNA vaccine components including the Kozak sequence, 5’ UTR, 3’UTR, Cap have been added with proper references. Our intention was to design a multi-epitope vaccine construct with features enabling both *in-silico* evaluation and potential experimental validation through recombinant expression. The cloning and codon optimization steps were included primarily to facilitate future laboratory-scale expression, purification, and characterization of the designed construct. Nevertheless, these steps are not definitive but predictive which requires experimental validation. The experimental validation is required has been added in detailed on the discussion as a drawback of this study.

2. Docking cluster scores appear to be interpreted as direct binding energies. In tools such as ClusPro, these scores are typically intended as relative ranking metrics rather than quantitative binding free energies. It would be helpful if the authors clarified how these values were interpreted. o “...strong binding affinities (-1126.8 kJ/mol) and (-1239.4 kJ/mol)” (lines 36-37)

**Response:** This section has been modified in the abstract and result of the revised manuscript as “Notably, the energy scores by Cluspro 2.0 do not directly estimate the binding free energy, but rather utilized for relative ranking of most probable poses of the docked complexes. So, these scores are predictive rather than definitive”.

3. Some MM-GBSA values reported in the manuscript appear unusually large (e.g., GB = 6855 kcal/mol; ELE = −6768.36 kcal/mol) (lines 353–356). Additional clarification of the calculation parameters and interpretation of these values would help readers better understand the results.

**Response:** The MM-GBSA analysis was performed using the HawkDock server, which employs an AMBER-based force field combined with the Generalized Born (GB) implicit solvation model. In this framework, individual energy components such as electrostatic (ELE) and polar solvation (GB) terms. These components are not intended to be interpreted independently, as they substantially compensate each other. The section has been updated as “Although individual MM-GBSA energy components (e.g., electrostatic and polar solvation terms) exhibit large magnitudes, these values largely compensate each other; therefore, the relative binding free energies are considered more reliable indicators for comparing complex stability.”

4. The manuscript describes a molecular dynamics (MD) simulation performed using iMOD (section 3.13). However, iMOD primarily performs normal mode analysis rather than classical molecular dynamics simulation. Clarification of this terminology would improve methodological accuracy.

**Response:** The terminology has been updated in every section of the revised manuscript**.**

5. The Ramachandran statistics indicate that approximately 82% of residues fall within the most favored regions (lines 323–325). While this may be acceptable for preliminary models, higher percentages are generally preferred when the structure is subsequently used for docking and epitope analysis. Further refinement or discussion of model quality may therefore be beneficial.

**Response:** The Ramachandran statistics has been upgraded by using a different server and added in the revised manuscript as “The Ramachandran plot from Ramplot server illustrated that the maximum amino acid residues occupied the most favorable regions 91.86%, allowed regions and disallowed regions 7.21% and 0.93% respectively”.

6. The manuscript suggests that stronger correlations observed in covariance maps indicate a more effective complex (lines 365–366). Covariance analyses generally describe correlated motions rather than biological effectiveness, so additional clarification of this interpretation would be helpful.

**Response:** We agree that covariance maps primarily reflect correlated atomic or residue-level motions derived from normal mode analysis and do not directly measure biological “effectiveness” in a functional or immunological sense. In the revised manuscript, this issue has been solved as “Stronger correlated motions observed in the covariance maps suggest enhanced dynamic coupling within the complex, potentially contributing to its structural stability, rather than directly indicating biological effectiveness”

7. The 3D model is too low quality for downstream use (docking and epitope prediction:
o “C score (-1.14), RMSD (9.6 ± 4.6 Å)” (section 3.9)

**Response:** We acknowledge that the predicted 3D model shows a relatively modest confidence score (C-score = −1.14; estimated RMSD = 9.6 ± 4.6 Å). However, this outcome is commonly observed in multi-epitope vaccine constructs because such chimeric proteins are artificial fusion sequences composed of multiple epitopes, adjuvants, and linker regions that do not naturally occur as stable globular proteins. As a result, structure-prediction algorithms frequently generate comparatively lower confidence scores for these highly flexible and non-native constructs. However, the model was improved on GalaxyRefine and that resulted in 91.86% amino acid residues to the most favoured region and the Z score was estimated as -1.81 which meant high similarity to the experimental level protein models.

8. The manuscript draws conclusions that contradict its own data or rely on misinterpreted outputs. Examples: The manuscript describes a binding free energy of +450.49 kcal/mol (lines 476-477) as indicating strong binding affinity. Since positive ΔG values generally correspond to unfavorable binding, further clarification of the calculation or interpretation of these values would be useful.

**Response:** We agree there was a typing mistake. The issue has been resolved in the result of the revised manuscript.

9. The manuscript interprets C-ImmSim results as indicating “year-long immunity “(lines 428–429). While immune simulations can provide useful theoretical insights, they generally cannot directly predict the duration of immunity *in-vivo*. The interpretation of these results may therefore benefit from a more cautious discussion.

**Response:** The statement has been modified as “These results predict that this vaccine can probably induce various immune cells” in the revised manuscript.

10. The manuscript states that mRNA stability was validated using RNA fold and minimum free energy values. While RNA fold can provide useful predictions of RNA secondary structure, additional considerations are typically required when evaluating the stability of mRNA vaccine constructs.

**Response:** We agree that prediction of secondary structure and minimum free energy (MFE) using RNAfold alone is not sufficient to comprehensively evaluate the stability of an mRNA vaccine construct. In the current study, RNAfold analysis was primarily used as an initial computational approach to assess the thermodynamic favorability and secondary structural organization of the designed mRNA sequence. We acknowledge that mRNA stability is influenced by multiple additional factors, including GC content, codon optimization, untranslated regions (UTRs), nucleotide modifications, poly(A) tail length, secondary structural complexity near the ribosome binding/initiation region, susceptibility to nuclease degradation, and intracellular translational efficiency. Experimental validation approaches such as in vitro transcription assays, ribonuclease resistance studies, expression profiling, and cellular translation efficiency analyses are also important for comprehensive stability assessment. To address the reviewer’s concern, we have revised the discussion of the manuscript as “RNAfold-derived MFE values were used as a preliminary in silico indicator of structural stability rather than definitive proof of biological stability.”
**Reviewer #2: Questions**

1 The introduction is well written, but it would benefit from adding more recent references (2024–2025) on Oropouche virus epidemiology and vaccine development.

**Response:** The more recent references have been added in the introduction of the revised manuscript.

2. The manuscript should clearly explain why the selected epitopes were prioritized, especially when multiple candidates were available.

**Response:** This fact has been added in the discussion section of the revised manuscript as “Notably, the epitopes were selected based on best immunological properties (antigenicity, non-allergenicity and non-toxicity), lowest possible percentile rank (<1.00) and high population coverage.”

3 Some figures need clearer labeling and higher resolution to improve readability.

**Response:** The problematic figures were replaced with higher resolution.

4 The manuscript contains minor grammatical and typographical errors that should be corrected during revision.

**Response:** The grammatical and typographical errors have been corrected in the revised manuscript.

5 A short statement about future experimental validation (in vitro and in vivo studies) would strengthen the practical relevance of the work.

**Response:** A short statement has been added at the end of the discussion section as “As a result, validation of this work requires both preclinical and clinical trials with long term precise inspection by the regulatory bodies such as the Food and Drug Administrations of USA, Centers for Disease Control and Prevention etc.”

6 The study uses iMOD server for molecular dynamics simulation, which performs normal mode analysis (NMA) rather than classical MD simulations. The manuscript should clarify this distinction and avoid interpreting the results as full MD simulations performed using tools like GROMACS or AMBER.

**Response:** All the terminology and result interpretation have been corrected to normal mode analysis in all the steps of the revised manuscript.

7 The reported 100% global population coverage appears unusually high. The manuscript should provide: Detailed HLA allele distribution data. The list of specific alleles used for coverage calculation. This would allow readers to verify the reliability of the population coverage analysis.

**Response:** We agree that achieving a reported 100% global population coverage may appear unusually high and therefore requires clarification. The value was generated using the population coverage analysis tool implemented in IEDB Population Coverage Tool based on the combined set of selected epitopes and their predicted interactions with multiple widely distributed HLA alleles across different geographic and ethnic populations.

We acknowledge that computational population coverage estimates represent theoretical predictions derived from available HLA frequency datasets and do not necessarily indicate absolute real-world coverage of the entire global population. The reported value reflects the broad theoretical distribution of the selected HLA-binding epitopes within the database rather than guaranteed universal immunogenicity in all individuals. To avoid overinterpretation, we have revised the manuscript to clarify that the observed population coverage represents a predicted computational estimate based on currently available HLA allele frequency data. We have also modified the wording in the Results and Discussion sections to present the finding more cautiously as “However, the population coverage was entire computational prediction rather than definitive statement. It requires experimental validation.” Additionally, the HLA allele distribution data has been added in the newly added supplementary table 1.

8 The vaccine gene was optimized for *E. coli* expression, even though the study proposes an mRNA vaccine platform. This raises a conceptual inconsistency. Codon optimization for bacterial expression vectors is typically relevant for recombinant protein vaccines rather than mRNA vaccines.

**Response:** Our intention was to design a multi-epitope vaccine construct with features enabling both *in-silico* evaluation and potential experimental validation through recombinant expression. The cloning and codon optimization steps were included primarily to facilitate future laboratory-scale expression, purification, and characterization of the designed construct. Nevertheless, these steps are not definitive but predictive which requires experimental validation. The experimental validation is required has been added in detailed on the discussion as a drawback of this study.

9 The immune simulation was performed using default parameters in C-ImmSim, but the manuscript does not justify the selected: vaccination schedule, simulation steps, antigen dosage. Providing justification or sensitivity analysis would improve confidence in the predicted immune responses.

**Response:** These parameters were already added on the manuscript as “The vaccine was administered in three doses at four-week intervals prior to full vaccination. All simulation parameters retained their default values. The steps and volume were set to 1000 and 50, respectively, and the random seed was set to 12,345 by default.”

10 Although the discussion mentions similar approaches for viruses such as SARS-CoV-2 and Zika, the manuscript does not directly compare its results with previously published OROV vaccine or epitope prediction studies. Including such comparisons would help contextualize the novelty and significance of the proposed vaccine.

**Response:** In the discussion of the revised manuscript, the comparison with the previously published oropouche vaccine has been conducted.
