# Supplementary material for: An immunoinformatics-driven multiepitope subunit vaccine targeting Oropouche virus: Molecular docking, immune and dynamics simulation approaches
Source: PLoS One. 2026 Oct 9;21(10):e0360186. doi: 10.1371/journal.pone.0360186 (PMC13652280; doi:10.1371/journal.pone.0360186)
Supplement: Supplementary file 6 [file pone.0360186.s016.docx]

We sincerely thank the Reviewer for this valuable observation and for highlighting the conceptual inconsistency in our previous manuscript version. We agree that the original presentation could lead to confusion regarding the intended vaccine platform.

**Additional Editor Comments:**

The authors have made useful revisions, but a fundamental inconsistency remains: the manuscript describes an mRNA vaccine while employing a recombinant protein workflow (His-tag, pET-28a(+), *E. coli* codon optimization). The clarified intention to enable "recombinant protein expression" confirms the construct is a protein vaccine, not an mRNA vaccine. This conceptual disconnect undermines the study's core claim and must be resolved. Additionally, conclusions from normal mode analysis and docking overreach their methodological support, and immune simulation results remain purely theoretical. The authors should either redefine the platform accurately or amend the title and revise the manuscript accordingly. Major revision is required before publication can be considered.

**Response:** The mRNA vaccine terminology has been modified to multiepitope subunit vaccine in the title and everywhere. The immune simulation is theoretical in this study, and it has been addressed at the end of the discussion section as a limitation that in the near future, the immune simulation will be conducted *in-vitro* and *in-vivo*.

Reviewer's Responses to Questions

**Comments to the Author**

**Reviewer #1:**

1. Response to Previous Comments

The authors have addressed several terminology-related and presentation-related concerns. However, the principal scientific concerns remain only partially resolved. Most notably, the study continues to describe an mRNA vaccine while employing a workflow that includes:

* His-tag incorporation

* 50S ribosomal protein adjuvant design

* Codon optimization for *Escherichia coli* K12

* Cloning into pET-28a(+)

* Recombinant protein expression considerations

* Protein structure modeling and protein-receptor docking

The addition of mRNA-associated elements such as the Kozak sequence, UTRs, and cap structure does not fully resolve the conceptual inconsistency. The manuscript still appears to combine features of recombinant protein vaccine development and mRNA vaccine design without clearly defining the intended platform or explaining how these elements are integrated into a coherent vaccine-development strategy. Their clarification: “Our intention was to design a multi‑epitope vaccine construct with features enabling both in‑silico evaluation and potential experimental validation through recombinant expression" confirms that their construct is a protein vaccine not an mRNA vaccine. Further clarification is required.

**Response:** In response to this issue, we have thoroughly revised the manuscript and substituted the phrase "mRNA vaccine" with "multi-epitope subunit vaccine" across the title, abstract, main text, figures, tables, supplemental materials, and all pertinent sections. The updated text now uniformly characterizes the suggested design as a recombinant multi-epitope subunit vaccination. Consequently, all design elements—including the 50S ribosomal protein adjuvant, His-tag, codon optimization for *Escherichia coli* K12, cloning into pET-28a(+), recombinant protein expression strategy, three-dimensional structure prediction, molecular docking, molecular dynamics simulation, and immune simulation—are now delineated within the suitable context of a recombinant protein-based vaccine aimed at future experimental validation.

2. Technical Soundness and Support for Conclusions. At present, I do not believe the conclusions are fully supported by the data presented.

Major concerns include:

a) Vaccine Platform Definition

The manuscript continues to describe the construct as an mRNA vaccine while retaining extensive recombinant protein expression features. The authors should clearly define whether the study represents:

* a true mRNA vaccine construct,

* a recombinant multi-epitope protein vaccine,

* or a hybrid conceptual design intended for both applications.

**Response:** In order to resolve this issue, we have conducted a thorough revision of the manuscript, replacing the term "mRNA vaccine" with "multi-epitope subunit vaccine" in the title, abstract, main text, figures, tables, supplementary materials, and all other pertinent sections. The construct is consistently depicted as a recombinant multi-epitope subunit vaccine in the revised manuscript.

b) Docking Interpretation

Although the authors now acknowledge that ClusPro scores are ranking metrics rather than true binding free energies, the manuscript still uses docking outcomes as evidence of robust receptor engagement and innate immune activation. Such conclusions should be interpreted more cautiously.

**Response:** The word selection has been modified in the discussion and conclusion part of docking and also in every section of the revised manuscript. For example, in the discussion of the docking, it has been modified as “Conversely, our vaccine construct showed predicted stronger binding affinities, with docking scores of −1126.8 kJ/mol (Vaccine-TLR-2) and −1239.4 kJ/mol (Vaccine-TLR-4), indicating probable stronger binding potential.”

c) MM-GBSA Analysis

The explanation regarding compensation between electrostatic and solvation energy terms is appreciated. However, the reported MM-GBSA component values remain unusually large and would benefit from stronger methodological justification. Readers should be able to understand why these values are considered reliable within the context of the chosen software and force field.

**Response:** The revised publication includes an additional discussion that clarifies that the HawkDock server, which uses a well-established MM-GBSA protocol based on the AMBER force field to estimate protein-protein binding free energies, was used to do the MM-GBSA binding free energy calculations. In this model, there is a lot of back-and-forth between the polar solvation energy and the electrostatic interaction energy because they are both huge but have the opposite sign. The overall binding free energy (ΔGbind) is the physically significant parameter used to evaluate binding affinity, rather than interpreting the individual energy elements separately. Consistent with the underlying MM-GBSA methodology in HawkDock and described in multiple protein-protein interaction studies using the same computational approach, our analysis found rather substantial electrostatic and solvation energy components. Therefore, the overall MM-GBSA binding free energy, not the absolute amount of the individual energy components, is used to determine the binding affinity assessment's reliability.
d) Normal Mode Analysis

The terminology has been corrected appropriately. However, conclusions regarding molecular stability should remain limited to the scope of normal mode analysis and should not imply the level of evidence typically obtained from classical molecular dynamics simulations.

**Response:** The normal mode analysis (NMA) offers merely a fundamental evaluation of collective molecular movements and should not be regarded as offering equivalent evidence to standard molecular dynamics (MD) simulations. In response to this issue, we have altered the language to guarantee that the conclusions derived from NMA are confined to its designated parameters, specifically the assessment of collective movements and inherent structural pliability. Moreover, we conducted a 100 ns classical molecular dynamics simulation of the vaccine and vaccine–TLR4 complex with GROMACS.

e) Structural Modeling

The improvement in Ramachandran statistics is noted and appreciated. Nevertheless, the construct remains an artificial fusion protein composed of multiple epitopes, linkers, and adjuvant sequences. The limitations of structure prediction and docking for such highly engineered constructs should be discussed more explicitly.

**Response:** This limitation has been added at the end of the discussion as “The modelled three-dimensional conformation demonstrated satisfactory stereochemical integrity; however, the suggested vaccine is a synthetically designed fusion protein that includes various epitopes, linkers, an adjuvant, and supplementary functional sequences. Thus, the predicted structure should to be considered as a computational prediction rather than an absolute representation of the natural protein configuration. Similarly, the outcomes of molecular docking rely on the precision of the anticipated structure and are unable to entirely consider conformational flexibility, protein folding, or dynamic molecular interactions in physiological environments. Consequently, although the structural modeling and docking studies offer significant first insights into the prospective behavior of the vaccine construct, their results necessitate verification through experimental structural characterisation and biological validation.”

f) Immune Simulation

The revised interpretation is more balanced than in the previous version. However, immune simulation results remain theoretical predictions and should not be presented as evidence of actual vaccine efficacy or protective immunity.

**Response:** In this current study, the immune simulation is theoretical and in future analyses, it will be performed *in-vivo* and the lack of experimental validation is added as a limitation at the end of the revised manuscript.

3. Statistical Rigor

The manuscript remains largely dependent on deterministic computational outputs and does not include formal statistical analyses, uncertainty estimates, sensitivity analyses, or assessment of prediction robustness. While this limitation is common in immunoinformatic studies, the manuscript would benefit from a clearer discussion of the uncertainty associated with computational predictions and parameter selection.

**Response:** Using immunoinformatics to design vaccines depends on computer predictions, which means there is some methodological uncertainty. The prediction algorithms used in this study are based on computer models that have been trained and parameters that have already been set. Their results should be seen as probabilistic predictions rather than certain biological events. To make our analyses more reliable, we did not choose epitopes based on just one prediction algorithm or parameter. Instead, many different factors were used to thoroughly screen the candidate epitopes. These included antigenicity, MHC-binding affinity, allergenicity, toxicity, conservancy, and population coverage. Also, the chosen vaccine construct was tested by analyzing its physicochemical properties, structural validity, predicting structural B-cell epitopes, molecular docking, 100 ns molecular dynamics simulation, MM-GBSA binding free-energy analysis, and immune simulation. The fact that these different computer analyses all came up with positive results boosts faith in the suggested vaccine option. Still, we are fully aware that these computational methods cannot get rid of the uncertainty in predictions or replace validation through experiments. Our Discussion has been expanded to make it clear that the results reported are just computer models and that the proposed multi-epitope subunit vaccine's immunogenicity, structural behavior, and ability to protect must be confirmed through *in-vitro* and *in-vivo* studies.

4. Data Availability and Reproducibility

The manuscript states that all relevant data are available within the manuscript and supporting information. However, reproducibility would be improved by providing, where possible:

* Raw docking output files

* MM-GBSA result files

* RNAfold outputs

* Immune simulation output files

* Complete construct sequences and supplementary datasets used in downstream analyses

The current level of data reporting may not be sufficient for full independent reproduction of all analyses.

**Response:** The raw docking output files, MM-GBSA result files, RNAfold outputs, immune simulation output files and complete construct sequence have been uploaded as a zipped file named “More Files for Reproducibility”.

5. Presentation and Language

The manuscript has improved compared with the previous version. Nevertheless, additional editing is recommended to improve:

* Scientific precision

* Consistency of terminology

* Sentence structure and readability

* Consistent distinction between mRNA vaccine and recombinant protein vaccine concepts

**Response:** The scientific precision, consistency of terminology, sentence structure and readability have been improved in the revised version of the manuscript and also the vaccine platform has been modified as multiepitope subunit vaccine.

**Reviewer #2:**

1. Can the predicted multi-epitope mRNA vaccine induce protective immunity against OROV in vivo?

**Response:** The current computational results indicate that the suggested vaccine may elicit protective immune responses. The incorporation of MHC class I, MHC class II, and B-cell epitopes could facilitate the probable stimulation of cytotoxic T lymphocytes, helper T cells, and antibody-secreting B cells. Furthermore, the predicted interactions with TLR2 and TLR4, the structural integrity noted throughout the 100 ns molecular dynamics simulation, and the immune simulation outcomes comprising predicted elevated antibody concentrations, IFN-γ synthesis, and memory cell proliferation—conclusively endorse its prospective immunogenicity. Nonetheless, these computational results do not validate that the vaccine will confer protective immunity against OROV *in-vivo*. The protective effectiveness must be determined using experimental methods involving recombinant expression and purification of the vaccine, succeeded by investigations on immunogenicity, safety, dose optimization, and viral challenges in an appropriate animal model. Such investigations ought to evaluate the generation of neutralizing antibodies, T-cell activity, cytokine profiles, viral load, clinical manifestations, survival rates, and tissue pathology. Additionally, these limitations have been added at the end of the discussion section.

2. How accurate are immunoinformatics-based epitope predictions compared with experimentally validated epitopes

**Response:** Immunoinformatics-driven epitope prediction tools offer a dependable and affordable method for ranking prospective vaccine candidates; yet, they cannot entirely replace experimental verification. The majority of modern predictive algorithms are constructed and refined utilizing extensive databases of experimentally confirmed epitopes, allowing them to discern potential antigenic areas with satisfactory predicted accuracy. However, in the future this will be confirmed by experimental validation and has been also added in the discussion section.

3. Why was disulfide engineering not performed to improve the structural stability of the vaccine construct?

**Response:** In the revised manuscript, the disulfide engineering has been performed.

4. Which discontinuous B-cell epitopes are present on the 3D structure of the vaccine construct, and how might they contribute to humoral immunity?

**Response:** The analysis of discontinuous B-cell epitopes was already part of the methodology. This analysis is actually the same thing as structural B-cell epitopes, which were discussed about in the 3.10 section of the materials and method along with an image and more information about the epitopes.

5. Why was the adjuvant placed at the N-terminal position instead of the C-terminal position in the vaccine construct?

**Response:** This has been mentioned at the methodology of the vaccine construction as “The adjuvant was deliberately placed at the N-terminal region, as this configuration is frequently utilised in multi-epitope vaccine development and is confirmed by immunoinformatics research. Positioning the adjuvant at the N-terminus enables its first translation and maintains its structural differentiation from the subsequent epitope sections, thus promoting effective identification by pattern recognition receptors (PRRs), including Toll-like receptors, contingent upon the chosen adjuvant. This orientation is anticipated to augment the primary innate immune activation, hence fostering more robust adaptive immunological reactions. Furthermore, the positioning of the N-terminal, in conjunction with a suitable stiff linker (such as EAAAK), aids in reducing structural disruption between the adjuvant and the epitope sequences, hence maintaining the autonomous folding and biological efficacy of each element. This design approach has been extensively utilised in earlier computational multi-epitope vaccine research due to its tendency to enhance structural stability and immunogenicity.”

6. Why was molecular docking specifically performed with TLR4 receptor?

**Response:** We would like to clarify that molecular docking was not performed exclusively with TLR4. In our study, the vaccine construct was docked with both TLR2 (PDB ID: 2Z7X) and TLR4 (PDB ID: 3FXI) to comprehensively evaluate its potential interactions with innate immune receptors. TLR2 and TLR4 were selected because they are key pattern recognition receptors (PRRs) involved in the initiation of innate immune responses through the recognition of pathogen-associated molecular patterns (PAMPs). Activation of these receptors triggers downstream signaling pathways that stimulate the production of pro-inflammatory cytokines and promote the development of adaptive immunity. Evaluating interactions with both receptors therefore provides a broader assessment of the vaccine candidate's potential immunostimulatory properties.

7. How do different untranslated regions (UTRs) influence mRNA stability and translation efficiency?

**Response:** We would like to clarify that, after the revision of the manuscript, the suggested design is uniformly characterized as a multi-epitope subunit vaccine instead of an mRNA vaccine. Consequently, the formulation and enhancement of mRNA-targeted regulatory components, such as 5′ and 3′ untranslated regions (UTRs), have been excluded from the vaccine development approach outlined in this research.

8. What dosage and delivery system would be optimal for this mRNA vaccine candidate?

**Response:** First, we would want to clarify that the proposed design, after manuscript modification, is consistently defined as a recombinant multi-epitope subunit vaccine, not an mRNA vaccine. Therefore, mRNA specific delivery methods, such as lipid nanoparticles, are not directly suitable for the present vaccine candidate. The delivery system has been added in the discussion of the revised manuscript as “For a recombinant subunit vaccine, computer analysis alone cannot be used to identify reliably the appropriate dose and delivery formulation. Based on standard procedures for recombinant protein vaccines, the purified vaccine design might be first assessed using intramuscular or subcutaneous administration with an appropriate authorized adjuvant or delivery system. Possible formulations include aluminium-based adjuvants, oil-in-water emulsions or biodegradable nanoparticle-based delivery methods. These formulations may improve the stability of the antigen, its uptake by antigen-presenting cells, and the generation of both humoral and cellular immune responses.”

9. Why was a full 100 ns Molecular Dynamics (MD) simulation not performed to validate the stability of the vaccine–TLR complexes?

**Response:** We have implemented a classical molecular dynamics simulation of the vaccine and vaccine–TLR4 complex in GROMACS within 100 ns, and for that a new section has been added in the revised manuscript. The trajectory was assessed using solvent-accessible surface area, hydrogen-bond analyses, radius of gyration, and RMSD and RMSF.

10. Could classical MD simulation using GROMACS or AMBER provide more realistic dynamic behavior of the vaccine construct compared with Normal Mode Analysis?

**Response:** Classical molecular dynamics simulation evaluates time-dependent atomic behavior under an explicitly defined force field and simulated physiological environment, whereas normal mode analysis provides a simplified assessment of collective molecular motions around an energy-minimized structure. Using GROMACS, we conducted a classical MD simulation of the vaccine–TLR4 complex in 100 ns to address this concern. Throughout the trajectory, this simulation allowed us to investigate structural deviation, residue-level flexibility, compactness, solvent exposure, and hydrogen-bond behavior. The results indicated that the complex maintained a structural profile that was relatively stable throughout the simulation.

11. How would RMSD, RMSF, radius of gyration (Rg), SASA, and hydrogen bond analyses from MD simulations support the structural stability of the vaccine complex?

**Response:** The vaccine–TLR4 complex maintained relatively limited overall structural deviation, as evidenced by the RMSD analysis, with values ranging from approximately 0.4 to 1.0 nm during the 100 ns simulation. The apo, in contrast, exhibited a larger degree of structural variation. The majority of the complex's residues exhibited minimal fluctuations, while the larger movements were restricted to specific regions rather than extending throughout the entire structure, as indicated by the RMSF analysis. This suggests that the complex maintained its overall structural organization, despite the localized flexibility. The complex's overall compactness was maintained without substantial unfolding or structural expansion, as evidenced by the relatively constant Rg profile. In the same vein, the SASA profile's stability indicated that the solvent exposure remained consistent throughout the trajectory. The presence of interactions during the simulation was further demonstrated by hydrogen-bond analysis, which corroborates the persistent molecular association between the vaccine and TLR4.
Combined, these analyses suggest that the vaccine–TLR4 complex maintained overall conformational stability, compactness, controlled residue flexibility, consistent solvent exposure, and persistent molecular interactions throughout the 100 ns simulation. The revised Results and Discussion sections now include these findings.

12. Can MM-PBSA/MM-GBSA calculations from MD trajectories provide more accurate binding free energy estimations than docking scores alone?

**Response:** We agree that MM-PBSA/MM-GBSA calculations that are conducted using representative frames extracted from an equilibrated molecular dynamics trajectory provide a more comprehensive estimation of binding free energy than docking scores alone, as they account for conformational changes that occur during the simulation. However, the MM-GBSA module, which is integrated into the HawkDock server, was employed to estimate the binding free energy in the current study. This module calculates the binding free energy by analyzing the docked protein–protein complex, rather than MD trajectory snapshots. This method has been extensively employed to evaluate protein–protein interactions and offers a more comprehensive evaluation of binding affinity than docking scores alone by integrating molecular mechanics and solvation energy terms. Additionally, we conducted a 100 ns classical molecular dynamics simulation in GROMACS to independently assess the structural stability of the vaccine and vaccine–TLR4 complex through RMSD, RMSF, Rg, SASA, and hydrogen-bond analyses, and we supplemented the HawkDock MM-GBSA analysis. Together, these complementary analyses offer computational support for the vaccine and vaccine–TLR4 complex's favorable interaction and stability. Additionally, we have added the lack of trajectory based MMGBSA/MMPBSA as a limitation and discussed that these will be conducted in future.

13. How can clinical and epidemiological findings on Oropouche virus infections support the real-world relevance and translational potential of the proposed vaccine candidate?

**Response:** The real-world relevance and translational potential of the proposed vaccine candidate have been added in the conclusion of the revised manuscript as “Overall, the favorable computational findings improve the proposed vaccine candidate's translational potential. The favorable physicochemical features, together with successful probable codon optimization and *in-silico* cloning, suggest the viability of recombinant production for further experimental studies. Although these findings need to be validated *in-vitro* and *in-vivo*, they provide compelling evidence for developing this multi-epitope subunit vaccine as a possible preventive candidate against Oropouche virus.”
