# Supplementary material for: An immunoinformatics-driven multiepitope subunit vaccine targeting Oropouche virus: Molecular docking, immune and dynamics simulation approaches
Source: PLoS One. 2026 Oct 9;21(10):e0360186. doi: 10.1371/journal.pone.0360186 (PMC13652280; doi:10.1371/journal.pone.0360186)
Supplement: Peer review history

# Peer review: Decision Letter 0

**DOI:** 10.1371/journal.pone.0360186.r001

**Authors:** Shahina Akter

13 Apr 2026

Dear Dr.  Mahdeen,

Thank you for submitting your manuscript to PLOS ONE. After careful consideration, we feel that it has merit but does not fully meet PLOS ONE’s publication criteria as it currently stands. Therefore, we invite you to submit a revised version of the manuscript that addresses the points raised during the review process.

Please submit your revised manuscript by May 28 2026 11:59PM. If you will need more time than this to complete your revisions, please reply to this message or contact the journal office at plosone@plos.org. When you're ready to submit your revision, log on to https://www.editorialmanager.com/pone/ and select the 'Submissions Needing Revision' folder to locate your manuscript file.

- A letter that responds to each point raised by the academic editor and reviewer(s). You should upload this letter as a separate file labeled 'Response to Reviewers'.
- A marked-up copy of your manuscript that highlights changes made to the original version. You should upload this as a separate file labeled 'Revised Manuscript with Track Changes'.
- An unmarked version of your revised paper without tracked changes. You should upload this as a separate file labeled 'Manuscript'.

If you would like to make changes to your financial disclosure, please include your updated statement in your cover letter. Guidelines for resubmitting your figure files are available below the reviewer comments at the end of this letter.

If applicable, we recommend that you deposit your laboratory protocols in protocols.io to enhance the reproducibility of your results. Protocols.io assigns your protocol its own identifier (DOI) so that it can be cited independently in the future. For instructions see: https://journals.plos.org/plosone/s/submission-guidelines#loc-laboratory-protocols. Additionally, PLOS ONE offers an option for publishing peer-reviewed Lab Protocol articles, which describe protocols hosted on protocols.io. Read more information on sharing protocols at https://plos.org/protocols?utm_medium=editorial-email&utm_source=authorletters&utm_campaign=protocols.

As the corresponding author, your ORCID iD is verified in the submission system and will appear in the published article. PLOS supports the use of ORCID, and we encourage all coauthors to register for an ORCID iD and use it as well. Please encourage your coauthors to verify their ORCID iD within the submission system before final acceptance, as unverified ORCID iDs will not appear in the published article. *Only*  the individual author can complete the verification step; PLOS staff *cannot*  verify ORCID iDs on behalf of authors.

We look forward to receiving your revised manuscript.

Kind regards,

Shahina Akter, Ph.D.

Academic Editor

PLOS One

Journal Requirements:

When submitting your revision, we need you to address these additional requirements.

1.Please ensure that your manuscript meets PLOS ONE's style requirements, including those for file naming. The PLOS ONE style templates can be found at

https://journals.plos.org/plosone/s/file?id=wjVg/PLOSOne_formatting_sample_main_body.pdf and

https://journals.plos.org/plosone/s/file?id=ba62/PLOSOne_formatting_sample_title_authors_affiliations.pdf

2. Please note that PLOS One has specific guidelines on code sharing for submissions in which author-generated code underpins the findings in the manuscript. In these cases, we expect all author-generated code to be made available without restrictions upon publication of the work. Please review our guidelines at https://journals.plos.org/plosone/s/materials-and-software-sharing#loc-sharing-code and ensure that your code is shared in a way that follows best practice and facilitates reproducibility and reuse.

3. When completing the data availability statement of the submission form, you indicated that you will make your data available on acceptance. We strongly recommend all authors decide on a data sharing plan before acceptance, as the process can be lengthy and hold up publication timelines. Please note that, though access restrictions are acceptable now, your entire data will need to be made freely accessible if your manuscript is accepted for publication. This policy applies to all data except where public deposition would breach compliance with the protocol approved by your research ethics board. If you are unable to adhere to our open data policy, please kindly revise your statement to explain your reasoning and we will seek the editor's input on an exemption. Please be assured that, once you have provided your new statement, the assessment of your exemption will not hold up the peer review process.

4. We note you have included a table to which you do not refer in the text of your manuscript. Please ensure that you refer to Table 2 in your text; if accepted, production will need this reference to link the reader to the Table.

5. Please include captions for your Supporting Information files at the end of your manuscript, and update any in-text citations to match accordingly. Please see our Supporting Information guidelines for more information: http://journals.plos.org/plosone/s/supporting-information.

6. If the reviewer comments include a recommendation to cite specific previously published works, please review and evaluate these publications to determine whether they are relevant and should be cited. There is no requirement to cite these works unless the editor has indicated otherwise.

[Note: HTML markup is below. Please do not edit.]

Reviewer's Responses to Questions

**Comments to the Author**

1. Is the manuscript technically sound, and do the data support the conclusions?

Reviewer #1: No

Reviewer #2: Yes

**********

2. Has the statistical analysis been performed appropriately and rigorously?

Reviewer #1: I Don't Know

Reviewer #2: Yes

**********

3. Have the authors made all data underlying the findings in their manuscript fully available?

The PLOS Data policy

Reviewer #1: No

Reviewer #2: Yes

**********

4. Is the manuscript presented in an intelligible fashion and written in standard English?

Reviewer #1: Yes

Reviewer #2: Yes

**********

Reviewer #1: General Assessment

This manuscript presents an immunoinformatics workflow described as the design of a “novel mRNA vaccine” against Oropouche virus (OROV). While the topic is relevant, the manuscript raises several conceptual and methodological concerns, including possible misinterpretation of computational outputs, internal inconsistencies, and incomplete presentation of figures and data. In particular, the central claim that an mRNA vaccine was designed appears inconsistent with several methodological elements that correspond more closely to a recombinant protein expression workflow. Substantial clarification and revision are therefore required to ensure that the study is conceptually and methodologically sound.

1. Is the manuscript technically sound, and do the data support the conclusions?

At present, the manuscript raises several conceptual and methodological concerns that make it difficult to conclude that the study is technically sound.

Key issues:

• The manuscript describes the design of an mRNA vaccine; however, several aspects of the workflow appear more consistent with recombinant protein expression strategies. Examples include the addition of a His-tag (lines 159–160), the use of a bacterial adjuvant (50S ribosomal protein of M. tuberculosis, lines 156–157), codon optimization for E. coli (section 3.14, lines 408–413), and cloning into the pET-28a(+) vector (lines 414–415). These elements are typically associated with bacterial protein expression rather than mRNA vaccine design. Clarification of the intended vaccine platform would therefore be helpful

• Docking cluster scores appear to be interpreted as direct binding energies. In tools such as ClusPro, these scores are typically intended as relative ranking metrics rather than quantitative binding free energies. It would be helpful if the authors clarified how these values were interpreted.

o “...strong binding affinities (-1126.8 kJ/mol) and (-1239.4 kJ/mol)” (lines 36-37)

• Some MM-GBSA values reported in the manuscript appear unusually large (e.g., GB = 6855 kcal/mol; ELE = −6768.36 kcal/mol) (lines 353–356). Additional clarification of the calculation parameters and interpretation of these values would help readers better understand the results.

• The manuscript describes molecular dynamics (MD) simulation performed using iMOD (section 3.13). However, iMOD primarily performs normal mode analysis rather than classical molecular dynamics simulation. Clarification of this terminology would improve methodological accuracy.

• The Ramachandran statistics indicate that approximately 82% of residues fall within the most favored regions (lines 323–325). While this may be acceptable for preliminary models, higher percentages are generally preferred when the structure is subsequently used for docking and epitope analysis. Further refinement or discussion of model quality may therefore be beneficial.

• The manuscript suggests that stronger correlations observed in covariance maps indicate a more effective complex (lines 365–366). Covariance analyses generally describe correlated motions rather than biological effectiveness, so additional clarification of this interpretation would be helpful.

• The 3D model is too low quality for downstream use (docking and epitope prediction:

o “C score (-1.14), RMSD (9.6 ± 4.6 Å)” (section 3.9)

• The manuscript draws conclusions that contradict its own data or rely on misinterpreted outputs.

Examples

• The manuscript describes a binding free energy of +450.49 kcal/mol (lines 476-477) as indicating strong binding affinity. Since positive ΔG values generally correspond to unfavorable binding, further clarification of the calculation or interpretation of these values would be useful.

• The manuscript interprets C-ImmSim results as indicating “year-long immunity “(lines 428–429). While immune simulations can provide useful theoretical insights, they generally cannot directly predict the duration of immunity in vivo. The interpretation of these results may therefore benefit from a more cautious discussion.

• he manuscript states that mRNA stability was validated using RNA fold and minimum free energy values. While RNA fold can provide useful predictions of RNA secondary structure, additional considerations are typically required when evaluating the stability of mRNA vaccine constructs.

2. Has the statistical analysis been performed appropriately and rigorously?

Recommendation: Statistical analysis is not clearly described in the manuscript. Since several conclusions rely on computational predictions, inclusion of statistical validation or discussion of prediction confidence would strengthen the analysis.

Examples

• No statistical validation of epitope predictions

• No confidence intervals or error estimates

• No statistical comparison of docking clusters

• No statistical analysis of immune simulation outputs

The manuscript treats deterministic computational outputs as if they were statistically validated.

3. Have the authors made all data underlying the findings in their manuscript fully available?

Response: Several figures referenced in the manuscript appear to be missing in the submitted version, which makes it difficult to evaluate some of the reported results.

4. Is the manuscript presented in an intelligible fashion and written in standard English?

Recommendation: The manuscript would benefit from improvements in presentation and language clarity. In addition, several figures referenced in the text appear to be missing, which prevents full evaluation of the results. The manuscript contains:

• Grammatical errors: “However” and “Nevertheless” are sometimes used where there is no contrast between the sentence and the one before it.

• Contradictory statements:

o “GRAVY score: -0.432” (line 35 and Table 4) and “The GRAVY 0.432” (lines 300) These cannot both be correct.

• Broken references

o “Error! Reference source not found.” (lines 425–426)

• Incorrect terminology:

o “hormonal immunological response” (lines 497).

Overall Recommendation: MAJOR REVISION

Overall, the manuscript addresses an interesting and relevant topic but would benefit from substantial conceptual clarification and methodological revision. In particular, the intended vaccine platform should be clearly defined, interpretations of docking and MM-GBSA results should be reconsidered, and missing figures and references should be corrected. With these revisions, the study may become clearer and more methodologically robust.

Reviewer #2: 1 The introduction is well written, but it would benefit from adding more recent references (2024–2025) on Oropouche virus epidemiology and vaccine development.

2 The manuscript should clearly explain why the selected epitopes were prioritized, especially when multiple candidates were available.

3 Some figures need clearer labeling and higher resolution to improve readability.

4 The manuscript contains minor grammatical and typographical errors that should be corrected during revision.

5 A short statement about future experimental validation (in vitro and in vivo studies) would strengthen the practical relevance of the work.

6 The study uses iMOD server for molecular dynamics simulation, which performs normal mode analysis (NMA) rather than classical MD simulations. The manuscript should clarify this distinction and avoid interpreting the results as full MD simulations performed using tools like GROMACS or AMBER.

7 The reported 100% global population coverage appears unusually high. The manuscript should provide: Detailed HLA allele distribution data. The list of specific alleles used for coverage calculation. This would allow readers to verify the reliability of the population coverage analysis.

8 The vaccine gene was optimized for E. coli expression, even though the study proposes an mRNA vaccine platform. This raises a conceptual inconsistency. Codon optimization for bacterial expression vectors is typically relevant for recombinant protein vaccines rather than mRNA vaccines.

9 The immune simulation was performed using default parameters in C-ImmSim, but the manuscript does not justify the selected: vaccination schedule, simulation steps, antigen dosage. Providing justification or sensitivity analysis would improve confidence in the predicted immune responses.

10 Although the discussion mentions similar approaches for viruses such as SARS-CoV-2 and Zika, the manuscript does not directly compare its results with previously published OROV vaccine or epitope prediction studies. Including such comparisons would help contextualize the novelty and significance of the proposed vaccine.

**********

what does this mean?). If published, this will include your full peer review and any attached files.

If you choose “no”, your identity will remain anonymous but your review may still be made public.

**Do you want your identity to be public for this peer review?**  For information about this choice, including consent withdrawal, please see our Privacy Policy

Reviewer #1: No

Reviewer #2: No

**********

[NOTE: If reviewer comments were submitted as an attachment file, they will be attached to this email and accessible via the submission site. Please log into your account, locate the manuscript record, and check for the action link "View Attachments". If this link does not appear, there are no attachment files.]

To ensure your figures meet our technical requirements, please review our figure guidelines: https://journals.plos.org/plosone/s/figures

You may also use PLOS’s free figure tool, NAAS, to help you prepare publication quality figures: https://journals.plos.org/plosone/s/figures#loc-tools-for-figure-preparation.

NAAS will assess whether your figures meet our technical requirements by comparing each figure against our figure specifications.

# Author comment: Author response to Decision Letter 1

**DOI:** 10.1371/journal.pone.0360186.r002

19 May 2026

Dear respected editor and reviewers, we acknowledge your suggestions and we have addressed the suggestions in the revised manuscript. Your suggestions will help us improve the manuscript.

Journal Requirements

1. Please ensure that your manuscript meets PLOS ONE's style requirements, including those for file naming. The PLOS ONE style templates can be found at

https://journals.plos.org/plosone/s/file?id=wjVg/PLOSOne_formatting_sample_main_body.pdf and

https://journals.plos.org/plosone/s/file?id=ba62/PLOSOne_formatting_sample_title_authors_affiliations.pdf

Response: The manuscript has been checked for the requirements of PLOS ONE's style and it was found that it meets the requirements.

2. Please note that PLOS One has specific guidelines on code sharing for submissions in which author-generated code underpins the findings in the manuscript. In these cases, we expect all author-generated code to be made available without restrictions upon publication of the work. Please review our guidelines at https://journals.plos.org/plosone/s/materials-and-software-sharing#loc-sharing-code and ensure that your code is shared in a way that follows best practice and facilitates reproducibility and reuse.

Response: We have reviewed the PLOS ONE code-sharing guidelines and will ensure that all author-generated code underpinning the findings of this manuscript is made publicly available without restrictions upon publication.

3. When completing the data availability statement of the submission form, you indicated that you will make your data available on acceptance. We strongly recommend all authors decide on a data sharing plan before acceptance, as the process can be lengthy and hold up publication timelines. Please note that, though access restrictions are acceptable now, your entire data will need to be made freely accessible if your manuscript is accepted for publication. This policy applies to all data except where public deposition would breach compliance with the protocol approved by your research ethics board. If you are unable to adhere to our open data policy, please kindly revise your statement to explain your reasoning and we will seek the editor's input on an exemption. Please be assured that, once you have provided your new statement, the assessment of your exemption will not hold up the peer review process.

Response: Thank you for your clarification regarding the data availability policy. The data is already available within the manuscript and the supplementary information.

4. We note you have included a table to which you do not refer in the text of your manuscript. Please ensure that you refer to Table 2 in your text; if accepted, production will need this reference to link the reader to the Table.

Response: The Table 2 has been referred to the revised manuscript

5. Please include captions for your Supporting Information files at the end of your manuscript, and update any in-text citations to match accordingly. Please see our Supporting Information guidelines for more information: http://journals.plos.org/plosone/s/supporting-information.

Response: The captions for the Supporting Information files have been added at the end of the revised manuscript.

6. If the reviewer comments include a recommendation to cite specific previously published works, please review and evaluate these publications to determine whether they are relevant and should be cited. There is no requirement to cite these works unless the editor has indicated otherwise.

Response: Thank you for your clarification. No author has told us to cite any published articles.

Reviewer's Responses to Questions

Reviewer #1: Questions

1. The manuscript describes the design of an mRNA vaccine; however, several aspects of the workflow appear more consistent with recombinant protein expression strategies. Examples include the addition of a His-tag (lines 159–160), the use of a bacterial adjuvant (50S ribosomal protein of M. tuberculosis, lines 156–157), codon optimization for E. coli (section 3.14, lines 408–413), and cloning into the pET-28a(+) vector (lines 414–415). These elements are typically associated with bacterial protein expression rather than mRNA vaccine design. Clarification of the intended vaccine platform would therefore be helpful.

Response: Thank you for your valuable observation. We agree that several components of the workflow, including the inclusion of the His-tag, the use of the 50S ribosomal protein adjuvant, codon optimization for E. coli, and cloning into the pET-28a(+) vector, are commonly associated with recombinant protein expression systems rather than conventional mRNA vaccine platforms. However, in the methodology and image of the vaccine construction of the revised manuscript, the other essential mRNA vaccine components including the Kozak sequence, 5’ UTR, 3’UTR, Cap have been added with proper references. Our intention was to design a multi-epitope vaccine construct with features enabling both in-silico evaluation and potential experimental validation through recombinant expression. The cloning and codon optimization steps were included primarily to facilitate future laboratory-scale expression, purification, and characterization of the designed construct. Nevertheless, these steps are not definitive but predictive which requires experimental validation. The experimental validation is required has been added in detailed on the discussion as a drawback of this study.

2. Docking cluster scores appear to be interpreted as direct binding energies. In tools such as ClusPro, these scores are typically intended as relative ranking metrics rather than quantitative binding free energies. It would be helpful if the authors clarified how these values were interpreted. o “...strong binding affinities (-1126.8 kJ/mol) and (-1239.4 kJ/mol)” (lines 36-37)

Response: This section has been modified in the abstract and result of the revised manuscript as “Notably, the energy scores by Cluspro 2.0 do not directly estimate the binding free energy, but rather utilized for relative ranking of most probable poses of the docked complexes. So, these scores are predictive rather than definitive”.

3. Some MM-GBSA values reported in the manuscript appear unusually large (e.g., GB = 6855 kcal/mol; ELE = −6768.36 kcal/mol) (lines 353–356). Additional clarification of the calculation parameters and interpretation of these values would help readers better understand the results.

Response: The MM-GBSA analysis was performed using the HawkDock server, which employs an AMBER-based force field combined with the Generalized Born (GB) implicit solvation model. In this framework, individual energy components such as electrostatic (ELE) and polar solvation (GB) terms. These components are not intended to be interpreted independently, as they substantially compensate each other. The section has been updated as “Although individual MM-GBSA energy components (e.g., electrostatic and polar solvation terms) exhibit large magnitudes, these values largely compensate each other; therefore, the relative binding free energies are considered more reliable indicators for comparing complex stability.”

4. The manuscript describes a molecular dynamics (MD) simulation performed using iMOD (section 3.13). However, iMOD primarily performs normal mode analysis rather than classical molecular dynamics simulation. Clarification of this terminology would improve methodological accuracy.

Response: The terminology has been updated in every section of the revised manuscript.

5. The Ramachandran statistics indicate that approximately 82% of residues fall within the most favored regions (lines 323–325). While this may be acceptable for preliminary models, higher percentages are generally preferred when the structure is subsequently used for docking and epitope analysis. Further refinement or discussion of model quality may therefore be beneficial.

Response: The Ramachandran statistics has been upgraded by using a different server and added in the revised manuscript as “The Ramachandran plot from Ramplot server illustrated that the maximum amino acid residues occupied the most favorable regions 91.86%, allowed regions and disallowed regions 7.21% and 0.93% respectively”.

6. The manuscript suggests that stronger correlations observed in covariance maps indicate a more effective complex (lines 365–366). Covariance analyses generally describe correlated motions rather than biological effectiveness, so additional clarification of this interpretation would be helpful.

Response: We agree that covariance maps primarily reflect correlated atomic or residue-level motions derived from normal mode analysis and do not directly measure biological “effectiveness” in a functional or immunological sense. In the revised manuscript, this issue has been solved as “Stronger correlated motions observed in the covariance maps suggest enhanced dynamic coupling within the complex, potentially contributing to its structural stability, rather than directly indicating biological effectiveness”

7. The 3D model is too low quality for downstream use (docking and epitope prediction:

o “C score (-1.14), RMSD (9.6 ± 4.6 Å)” (section 3.9)

Response: We acknowledge that the predicted 3D model shows a relatively modest confidence score (C-score = −1.14; estimated RMSD = 9.6 ± 4.6 Å). However, this outcome is commonly observed in multi-epitope vaccine constructs because such chimeric proteins are artificial fusion sequences composed of multiple epitopes, adjuvants, and linker regions that do not naturally occur as stable globular proteins. As a result, structure-prediction algorithms frequently generate comparatively lower confidence scores for these highly flexible and non-native constructs. However, the model was improved on GalaxyRefine and that resulted in 91.86% amino acid residues to the most favoured region and the Z score was estimated as -1.81 which meant high similarity to the experimental level protein models.

8. The manuscript draws conclusions that contradict its own data or rely on misinterpreted outputs. Examples: The manuscript describes a binding free energy of +450.49 kcal/mol (lines 476-477) as indicating strong binding affinity. Since positive ΔG values generally correspond to unfavorable binding, further clarification of the calculation or interpretation of these values would be useful.

Response: We agree there was a typing mistake. The issue has been resolved in the result of the revised manuscript.

9. The manuscript interprets C-ImmSim results as indicating “year-long immunity “(lines 428–429). While immune simulations can provide useful theoretical insights, they generally cannot directly predict the duration of immunity in-vivo. The interpretation of these results may therefore benefit from a more cautious discussion.

Response: The statement has been modified as “These results predict that this vaccine can probably induce various immune cells” in the revised manuscript.

10. The manuscript states that mRNA stability was validated using RNA fold and minimum free energy values. While RNA fold can provide useful predictions of RNA secondary structure, additional considerations are typically required when evaluating the stability of mRNA vaccine constructs.

Response: We agree that prediction of secondary structure and minimum free energy (MFE) using RNAfold alone is not sufficient to comprehensively evaluate the stability of an mRNA vaccine construct. In the current study, RNAfold analysis was primarily used as an initial computational approach to assess the thermodynamic favorability and secondary structural organization of the designed mRNA sequence. We acknowledge that mRNA stability is influenced by multiple additional factors, including GC content, codon optimization, untranslated regions (UTRs), nucleotide modifications, poly(A) tail length, secondary structural complexity near the ribosome binding/initiation region, susceptibility to nuclease degradation, and intracellular translational efficiency. Experimental validation approaches such as in vitro transcription assays, ribonuclease resistance studies, expression profiling, and cellular translation efficiency analyses are also important for comprehensive stability assessment. To address the reviewer’s concern, we have revised the discussion of the manuscript as “RNAfold-derived MFE values were used as a preliminary in silico indicator of structural stability rather than definitive proof of biological stability.”

Reviewer #2: Questions

1 The introduction is well written, but it would benefit from adding more recent references (2024–2025) on Oropouche virus epidemiology and vaccine development.

Response: The more recent references have been added in the introduction of the revised manuscript.

2. The manuscript should clearly explain why the selected epitopes were prioritized, especially when multiple candidates were available.

Response: This fact has been added in the discussion section of the revised manuscript as “Notably, the epitopes were selected based on best immunological properties (antigenicity, non-allergenicity and non-toxicity), lowest possible percentile rank (<1.00) and high population coverage.”

3 Some figures need clearer labeling and higher resolution to improve readability.

Response: The problematic figures were replaced with higher resolution.

4 The manuscript contains minor grammatical and typographical errors that should be corrected during revision.

Response: The grammatical and typographical errors have been corrected in the revised manuscript.

5 A short statement about future experimental validation (in vitro and in vivo studies) would strengthen the practical relevance of the work.

Response: A short statement has been added at the end of the discussion section as “As a result, validation of this work requires both preclinical and clinical trials with long term precise inspection by the regulatory bodies such as the Food and Drug Administrations of USA, Centers for Disease Control and Prevention etc.”

6 The study uses iMOD server for molecular dynamics simulation, which performs normal mode analysis (NMA) rather than classical MD simulations. The manuscript should clarify this distinction and avoid interpreting the results as full MD simulations performed using tools like GROMACS or AMBER.

Response: All the terminology and result interpretation have been corrected to normal mode analysis in all the steps of the revised manuscript.

7 The reported 100% global population coverage appears unusually high. The manuscript should provide: Detailed HLA allele distribution data. The list of specific alleles used for coverage calculation. This would allow readers to verify the reliability of the population coverage analysis.

Response: We agree that achieving a reported 100% global population coverage may appear unusually high and therefore requires clarification. The value was generated using the population coverage analysis tool implemented in IEDB Population Coverage Tool based on the combined set of selected epitopes and their predicted interactions with multiple widely distributed HLA alleles across different geographic and ethnic populations.

We acknowledge that computational population coverage estimates represent theoretical predictions derived from available HLA frequency datasets and do not necessarily indicate absolute real-world coverage of the entire global population. The reported value reflects the broad theoretical distribution of the selected HLA-binding epitopes within the database rather than guaranteed universal immunogenicity in all individuals. To avoid overinterpretation, we have revised the manuscript to clarify that the observed population coverage represents a predicted computational estimate based on currently available HLA allele frequency data. We have also modified the wording in the Results and Discussion sections to present the finding more cautiously as “However, the population coverage was entire computational prediction rather than definitive statement. It requires experimental validation.” Additionally, the HLA allele distribution data has been added in the newly added supplementary table 1.

8 The vaccine gene was optimized for E. coli expression, even though the study proposes an mRNA vaccine platform. This raises a conceptual inconsistency. Codon optimization for bacterial expression vectors is typically relevant for recombinant protein vaccines rather than mRNA vaccines.

Response: Our intention was to design a multi-epitope vaccine construct with features enabling both in-silico evaluation and potential experimental validation through recombinant expression. The cloning and codon optimization steps were included primarily to facilitate future laboratory-scale expression, purification, and characterization of the designed construct. Nevertheless, these steps are not definitive but predictive which requires experimental validation. The experimental validation is required has been added in detailed on the discussion as a drawback of this study.

9 The immune simulation was performed using default parameters in C-ImmSim, but the manuscript does not justify the selected: vaccination schedule, simulation steps, antigen dosage. Providing justification or sensitivity analysis would improve confidence in the predicted immune responses.

Response: These parameters were already added on the manuscript as “The vaccine was administered in three doses at four-week intervals prior to full vaccination. All simulation parameters retained their default values. The steps and volume were set to 1000 and 50, respectively, and the random seed was set to 12,345 by default.”

10 Although the discussion mentions similar approaches for viruses such as SARS-CoV-2 and Zika, the manuscript does not directly compare its results with previously published OROV vaccine or epitope prediction studies. Including such comparisons would help contextualize the novelty and significance of the proposed vaccine.

Response: In the discussion of the revised manuscript, the comparison with the previously published oropouche vaccine has been conducted.

Attachment Submitted filename: Response to Reviewers.docx

# Peer review: Decision Letter 1

**DOI:** 10.1371/journal.pone.0360186.r003

**Authors:** Shahina Akter

12 Jun 2026

Dear Dr. Mahdeen,

Thank you for submitting your manuscript to PLOS ONE. After careful consideration, we feel that it has merit but does not fully meet PLOS ONE’s publication criteria as it currently stands. Therefore, we invite you to submit a revised version of the manuscript that addresses the points raised during the review process.

Please submit your revised manuscript by Jul 27 2026 11:59PM. If you will need more time than this to complete your revisions, please reply to this message or contact the journal office at plosone@plos.org. When you're ready to submit your revision, log on to https://www.editorialmanager.com/pone/ and select the 'Submissions Needing Revision' folder to locate your manuscript file.

- A letter that responds to each point raised by the academic editor and reviewer(s). You should upload this letter as a separate file labeled 'Response to Reviewers'.
- A marked-up copy of your manuscript that highlights changes made to the original version. You should upload this as a separate file labeled 'Revised Manuscript with Track Changes'.
- An unmarked version of your revised paper without tracked changes. You should upload this as a separate file labeled 'Manuscript'.

If you would like to make changes to your financial disclosure, please include your updated statement in your cover letter. Guidelines for resubmitting your figure files are available below the reviewer comments at the end of this letter.

If applicable, we recommend that you deposit your laboratory protocols in protocols.io to enhance the reproducibility of your results. Protocols.io assigns your protocol its own identifier (DOI) so that it can be cited independently in the future. For instructions see: https://journals.plos.org/plosone/s/submission-guidelines#loc-laboratory-protocols. Additionally, PLOS ONE offers an option for publishing peer-reviewed Lab Protocol articles, which describe protocols hosted on protocols.io. Read more information on sharing protocols at https://plos.org/protocols?utm_medium=editorial-email&utm_source=authorletters&utm_campaign=protocols.

As the corresponding author, your ORCID iD is verified in the submission system and will appear in the published article. PLOS supports the use of ORCID, and we encourage all coauthors to register for an ORCID iD and use it as well. Please encourage your coauthors to verify their ORCID iD within the submission system before final acceptance, as unverified ORCID iDs will not appear in the published article. *Only*  the individual author can complete the verification step; PLOS staff *cannot*  verify ORCID iDs on behalf of authors.

We look forward to receiving your revised manuscript.

Kind regards,

Shahina Akter, Ph.D.

Academic Editor

PLOS One

Journal Requirements:

If the reviewer comments include a recommendation to cite specific previously published works, please review and evaluate these publications to determine whether they are relevant and should be cited. There is no requirement to cite these works unless the editor has indicated otherwise.

**Additional Editor Comments:**

The authors have made useful revisions, but a fundamental inconsistency remains: the manuscript describes an mRNA vaccine while employing a recombinant protein workflow (His-tag, pET-28a(+), E. coli codon optimization). The clarified intention to enable "recombinant protein expression" confirms the construct is a protein vaccine, not an mRNA vaccine. This conceptual disconnect undermines the study's core claim and must be resolved. Additionally, conclusions from normal mode analysis and docking overreach their methodological support, and immune simulation results remain purely theoretical. The authors should either redefine the platform accurately or amend the title and revise the manuscript accordingly. Major revision is required before publication can be considered.

[Note: HTML markup is below. Please do not edit.]

Reviewers' comments:

Reviewer's Responses to Questions

**Comments to the Author**

Reviewer #1: (No Response)

Reviewer #2: (No Response)

**********

2. Is the manuscript technically sound, and do the data support the conclusions?

Reviewer #1: No

Reviewer #2: Yes

**********

3. Has the statistical analysis been performed appropriately and rigorously?

Reviewer #1: No

Reviewer #2: Yes

**********

4. Have the authors made all data underlying the findings in their manuscript fully available?

The PLOS Data policy

Reviewer #1: No

Reviewer #2: Yes

**********

5. Is the manuscript presented in an intelligible fashion and written in standard English?

Reviewer #1: Yes

Reviewer #2: Yes

**********

Reviewer #1: Overall Assessment

The authors have made a number of revisions in response to the previous review, including clarification of the distinction between normal mode analysis and molecular dynamics simulation, improvement of the reported Ramachandran statistics, more cautious interpretation of immune simulation results, clarification of the limitations of RNAfold-derived stability predictions, and acknowledgment that ClusPro scores are relative ranking metrics rather than direct binding free energies.

These revisions improve the manuscript. However, several major conceptual and methodological concerns remain unresolved. Most importantly, the central inconsistency between the stated mRNA vaccine platform and the predominantly recombinant protein-based workflow remains insufficiently addressed. In addition, several computational analyses continue to be interpreted more strongly than warranted by the underlying methods.

Consequently, I do not believe the manuscript is yet ready for publication in its current form.

1. Response to Previous Comments

The authors have addressed several terminology-related and presentation-related concerns. However, the principal scientific concerns remain only partially resolved.

Most notably, the study continues to describe an mRNA vaccine while employing a workflow that includes:

* His-tag incorporation

* 50S ribosomal protein adjuvant design

* Codon optimization for Escherichia coli K12

* Cloning into pET-28a(+)

* Recombinant protein expression considerations

* Protein structure modeling and protein-receptor docking

The addition of mRNA-associated elements such as the Kozak sequence, UTRs, and cap structure does not fully resolve the conceptual inconsistency. The manuscript still appears to combine features of recombinant protein vaccine development and mRNA vaccine design without clearly defining the intended platform or explaining how these elements are integrated into a coherent vaccine-development strategy.

Their clarification: “Our intention was to design a multi‑epitope vaccine construct with features enabling both in‑silico evaluation and potential experimental validation through recombinant expression" confirms that their construct is a protein vaccine not an mRNA vaccine.

Further clarification is required.

2. Technical Soundness and Support for Conclusions

At present, I do not believe the conclusions are fully supported by the data presented.

Major concerns include:

a) Vaccine Platform Definition

The manuscript continues to describe the construct as an mRNA vaccine while retaining extensive recombinant protein expression features. The authors should clearly define whether the study represents:

* a true mRNA vaccine construct,

* a recombinant multi-epitope protein vaccine,

* or a hybrid conceptual design intended for both applications.

b) Docking Interpretation

Although the authors now acknowledge that ClusPro scores are ranking metrics rather than true binding free energies, the manuscript still uses docking outcomes as evidence of robust receptor engagement and innate immune activation. Such conclusions should be interpreted more cautiously.

c) MM-GBSA Analysis

The explanation regarding compensation between electrostatic and solvation energy terms is appreciated. However, the reported MM-GBSA component values remain unusually large and would benefit from stronger methodological justification. Readers should be able to understand why these values are considered reliable within the context of the chosen software and force field.

d) Normal Mode Analysis

The terminology has been corrected appropriately. However, conclusions regarding molecular stability should remain limited to the scope of normal mode analysis and should not imply the level of evidence typically obtained from classical molecular dynamics simulations.

e) Structural Modeling

The improvement in Ramachandran statistics is noted and appreciated. Nevertheless, the construct remains an artificial fusion protein composed of multiple epitopes, linkers, and adjuvant sequences. The limitations of structure prediction and docking for such highly engineered constructs should be discussed more explicitly.

f) Immune Simulation

The revised interpretation is more balanced than in the previous version. However, immune simulation results remain theoretical predictions and should not be presented as evidence of actual vaccine efficacy or protective immunity.

3. Statistical Rigor

The manuscript remains largely dependent on deterministic computational outputs and does not include formal statistical analyses, uncertainty estimates, sensitivity analyses, or assessment of prediction robustness.

While this limitation is common in immunoinformatic studies, the manuscript would benefit from a clearer discussion of the uncertainty associated with computational predictions and parameter selection.

4. Data Availability and Reproducibility

The manuscript states that all relevant data are available within the manuscript and supporting information. However, reproducibility would be improved by providing, where possible:

* Raw docking output files

* MM-GBSA result files

* RNAfold outputs

* Immune simulation output files

* Complete construct sequences and supplementary datasets used in downstream analyses

The current level of data reporting may not be sufficient for full independent reproduction of all analyses.

5. Presentation and Language

The manuscript has improved compared with the previous version. Nevertheless, additional editing is recommended to improve:

* Scientific precision

* Consistency of terminology

* Sentence structure and readability

* Consistent distinction between mRNA vaccine and recombinant protein vaccine concepts

Recommendation

Major Revision

The manuscript addresses a relevant and timely topic and has improved following revision. However, important conceptual and methodological concerns remain unresolved, particularly regarding the definition and implementation of the proposed vaccine platform.

Before publication, the authors should provide a clearer and scientifically consistent description of the vaccine strategy, further moderate interpretations of computational results, and strengthen methodological transparency and reproducibility. The authors could consider amending the title of the work to reflect the appropriate vaccine platform.

Addressing these issues would substantially improve the scientific clarity and reliability of the study.

Reviewer #2: 1. Can the predicted multi-epitope mRNA vaccine induce protective immunity against OROV in vivo?

2. How accurate are immunoinformatics-based epitope predictions compared with experimentally validated epitopes?

3. Why was disulfide engineering not performed to improve the structural stability of the vaccine construct?

4. Which discontinuous B-cell epitopes are present on the 3D structure of the vaccine construct, and how might they contribute to humoral immunity?

5. Why was the adjuvant placed at the N-terminal position instead of the C-terminal position in the vaccine construct?

6. Why was molecular docking specifically performed with TLR4 receptor?

7. How do different untranslated regions (UTRs) influence mRNA stability and translation efficiency?

8. What dosage and delivery system would be optimal for this mRNA vaccine candidate?

9. Why was a full 100 ns Molecular Dynamics (MD) simulation not performed to validate the stability of the vaccine–TLR complexes?

10. Could classical MD simulation using GROMACS or AMBER provide more realistic dynamic behavior of the vaccine construct compared with Normal Mode Analysis?

11. How would RMSD, RMSF, radius of gyration (Rg), SASA, and hydrogen bond analyses from MD simulations support the structural stability of the vaccine complex?

12. Can MM-PBSA/MM-GBSA calculations from MD trajectories provide more accurate binding free energy estimations than docking scores alone?

13. How can clinical and epidemiological findings on Oropouche virus infections support the real-world relevance and translational potential of the proposed vaccine candidate?

**********

what does this mean?). If published, this will include your full peer review and any attached files.

If you choose “no”, your identity will remain anonymous but your review may still be made public.

**Do you want your identity to be public for this peer review?**  For information about this choice, including consent withdrawal, please see our Privacy Policy

Reviewer #1: No

Reviewer #2: No

**********

[NOTE: If reviewer comments were submitted as an attachment file, they will be attached to this email and accessible via the submission site. Please log into your account, locate the manuscript record, and check for the action link "View Attachments". If this link does not appear, there are no attachment files.]

To ensure your figures meet our technical requirements, please review our figure guidelines: https://journals.plos.org/plosone/s/figures

You may also use PLOS’s free figure tool, NAAS, to help you prepare publication quality figures: https://journals.plos.org/plosone/s/figures#loc-tools-for-figure-preparation.

NAAS will assess whether your figures meet our technical requirements by comparing each figure against our figure specifications.

# Author comment: Author response to Decision Letter 2

**DOI:** 10.1371/journal.pone.0360186.r004

6 Aug 2026

We sincerely thank the Reviewer for this valuable observation and for highlighting the conceptual inconsistency in our previous manuscript version. We agree that the original presentation could lead to confusion regarding the intended vaccine platform.

Additional Editor Comments:

The authors have made useful revisions, but a fundamental inconsistency remains: the manuscript describes an mRNA vaccine while employing a recombinant protein workflow (His-tag, pET-28a(+), E. coli codon optimization). The clarified intention to enable "recombinant protein expression" confirms the construct is a protein vaccine, not an mRNA vaccine. This conceptual disconnect undermines the study's core claim and must be resolved. Additionally, conclusions from normal mode analysis and docking overreach their methodological support, and immune simulation results remain purely theoretical. The authors should either redefine the platform accurately or amend the title and revise the manuscript accordingly. Major revision is required before publication can be considered.

Response: The mRNA vaccine terminology has been modified to multiepitope subunit vaccine in the title and everywhere. The immune simulation is theoretical in this study, and it has been addressed at the end of the discussion section as a limitation that in the near future, the immune simulation will be conducted in-vitro and in-vivo.

Reviewer's Responses to Questions

Comments to the Author

Reviewer #1:

1. Response to Previous Comments

The authors have addressed several terminology-related and presentation-related concerns. However, the principal scientific concerns remain only partially resolved. Most notably, the study continues to describe an mRNA vaccine while employing a workflow that includes:

* His-tag incorporation

* 50S ribosomal protein adjuvant design

* Codon optimization for Escherichia coli K12

* Cloning into pET-28a(+)

* Recombinant protein expression considerations

* Protein structure modeling and protein-receptor docking

The addition of mRNA-associated elements such as the Kozak sequence, UTRs, and cap structure does not fully resolve the conceptual inconsistency. The manuscript still appears to combine features of recombinant protein vaccine development and mRNA vaccine design without clearly defining the intended platform or explaining how these elements are integrated into a coherent vaccine-development strategy. Their clarification: “Our intention was to design a multi epitope vaccine construct with features enabling both in silico evaluation and potential experimental validation through recombinant expression" confirms that their construct is a protein vaccine not an mRNA vaccine. Further clarification is required.

Response: In response to this issue, we have thoroughly revised the manuscript and substituted the phrase "mRNA vaccine" with "multi-epitope subunit vaccine" across the title, abstract, main text, figures, tables, supplemental materials, and all pertinent sections. The updated text now uniformly characterizes the suggested design as a recombinant multi-epitope subunit vaccination. Consequently, all design elements—including the 50S ribosomal protein adjuvant, His-tag, codon optimization for Escherichia coli K12, cloning into pET-28a(+), recombinant protein expression strategy, three-dimensional structure prediction, molecular docking, molecular dynamics simulation, and immune simulation—are now delineated within the suitable context of a recombinant protein-based vaccine aimed at future experimental validation.

2. Technical Soundness and Support for Conclusions. At present, I do not believe the conclusions are fully supported by the data presented.

Major concerns include:

a) Vaccine Platform Definition

The manuscript continues to describe the construct as an mRNA vaccine while retaining extensive recombinant protein expression features. The authors should clearly define whether the study represents:

* a true mRNA vaccine construct,

* a recombinant multi-epitope protein vaccine,

* or a hybrid conceptual design intended for both applications.

Response: In order to resolve this issue, we have conducted a thorough revision of the manuscript, replacing the term "mRNA vaccine" with "multi-epitope subunit vaccine" in the title, abstract, main text, figures, tables, supplementary materials, and all other pertinent sections. The construct is consistently depicted as a recombinant multi-epitope subunit vaccine in the revised manuscript.

b) Docking Interpretation

Although the authors now acknowledge that ClusPro scores are ranking metrics rather than true binding free energies, the manuscript still uses docking outcomes as evidence of robust receptor engagement and innate immune activation. Such conclusions should be interpreted more cautiously.

Response: The word selection has been modified in the discussion and conclusion part of docking and also in every section of the revised manuscript. For example, in the discussion of the docking, it has been modified as “Conversely, our vaccine construct showed predicted stronger binding affinities, with docking scores of −1126.8 kJ/mol (Vaccine-TLR-2) and −1239.4 kJ/mol (Vaccine-TLR-4), indicating probable stronger binding potential.”

c) MM-GBSA Analysis

The explanation regarding compensation between electrostatic and solvation energy terms is appreciated. However, the reported MM-GBSA component values remain unusually large and would benefit from stronger methodological justification. Readers should be able to understand why these values are considered reliable within the context of the chosen software and force field.

Response: The revised publication includes an additional discussion that clarifies that the HawkDock server, which uses a well-established MM-GBSA protocol based on the AMBER force field to estimate protein-protein binding free energies, was used to do the MM-GBSA binding free energy calculations. In this model, there is a lot of back-and-forth between the polar solvation energy and the electrostatic interaction energy because they are both huge but have the opposite sign. The overall binding free energy (ΔGbind) is the physically significant parameter used to evaluate binding affinity, rather than interpreting the individual energy elements separately. Consistent with the underlying MM-GBSA methodology in HawkDock and described in multiple protein-protein interaction studies using the same computational approach, our analysis found rather substantial electrostatic and solvation energy components. Therefore, the overall MM-GBSA binding free energy, not the absolute amount of the individual energy components, is used to determine the binding affinity assessment's reliability.

d) Normal Mode Analysis

The terminology has been corrected appropriately. However, conclusions regarding molecular stability should remain limited to the scope of normal mode analysis and should not imply the level of evidence typically obtained from classical molecular dynamics simulations.

Response: The normal mode analysis (NMA) offers merely a fundamental evaluation of collective molecular movements and should not be regarded as offering equivalent evidence to standard molecular dynamics (MD) simulations. In response to this issue, we have altered the language to guarantee that the conclusions derived from NMA are confined to its designated parameters, specifically the assessment of collective movements and inherent structural pliability. Moreover, we conducted a 100 ns classical molecular dynamics simulation of the vaccine and vaccine–TLR4 complex with GROMACS.

e) Structural Modeling

The improvement in Ramachandran statistics is noted and appreciated. Nevertheless, the construct remains an artificial fusion protein composed of multiple epitopes, linkers, and adjuvant sequences. The limitations of structure prediction and docking for such highly engineered constructs should be discussed more explicitly.

Response: This limitation has been added at the end of the discussion as “The modelled three-dimensional conformation demonstrated satisfactory stereochemical integrity; however, the suggested vaccine is a synthetically designed fusion protein that includes various epitopes, linkers, an adjuvant, and supplementary functional sequences. Thus, the predicted structure should to be considered as a computational prediction rather than an absolute representation of the natural protein configuration. Similarly, the outcomes of molecular docking rely on the precision of the anticipated structure and are unable to entirely consider conformational flexibility, protein folding, or dynamic molecular interactions in physiological environments. Consequently, although the structural modeling and docking studies offer significant first insights into the prospective behavior of the vaccine construct, their results necessitate verification through experimental structural characterisation and biological validation.”

f) Immune Simulation

The revised interpretation is more balanced than in the previous version. However, immune simulation results remain theoretical predictions and should not be presented as evidence of actual vaccine efficacy or protective immunity.

Response: In this current study, the immune simulation is theoretical and in future analyses, it will be performed in-vivo and the lack of experimental validation is added as a limitation at the end of the revised manuscript.

3. Statistical Rigor

The manuscript remains largely dependent on deterministic computational outputs and does not include formal statistical analyses, uncertainty estimates, sensitivity analyses, or assessment of prediction robustness. While this limitation is common in immunoinformatic studies, the manuscript would benefit from a clearer discussion of the uncertainty associated with computational predictions and parameter selection.

Response: Using immunoinformatics to design vaccines depends on computer predictions, which means there is some methodological uncertainty. The prediction algorithms used in this study are based on computer models that have been trained and parameters that have already been set. Their results should be seen as probabilistic predictions rather than certain biological events. To make our analyses more reliable, we did not choose epitopes based on just one prediction algorithm or parameter. Instead, many different factors were used to thoroughly screen the candidate epitopes. These included antigenicity, MHC-binding affinity, allergenicity, toxicity, conservancy, and population coverage. Also, the chosen vaccine construct was tested by analyzing its physicochemical properties, structural validity, predicting structural B-cell epitopes, molecular docking, 100 ns molecular dynamics simulation, MM-GBSA binding free-energy analysis, and immune simulation. The fact that these different computer analyses all came up with positive results boosts faith in the suggested vaccine option. Still, we are fully aware that these computational methods cannot get rid of the uncertainty in predictions or replace validation through experiments. Our Discussion has been expanded to make it clear that the results reported are just computer models and that the proposed multi-epitope subunit vaccine's immunogenicity, structural behavior, and ability to protect must be confirmed through in-vitro and in-vivo studies.

4. Data Availability and Reproducibility

The manuscript states that all relevant data are available within the manuscript and supporting information. However, reproducibility would be improved by providing, where possible:

* Raw docking output files

* MM-GBSA result files

* RNAfold outputs

* Immune simulation output files

* Complete construct sequences and supplementary datasets used in downstream analyses

The current level of data reporting may not be sufficient for full independent reproduction of all analyses.

Response: The raw docking output files, MM-GBSA result files, RNAfold outputs, immune simulation output files and complete construct sequence have been uploaded as a zipped file named “More Files for Reproducibility”.

5. Presentation and Language

The manuscript has improved compared with the previous version. Nevertheless, additional editing is recommended to improve:

* Scientific precision

* Consistency of terminology

* Sentence structure and readability

* Consistent distinction between mRNA vaccine and recombinant protein vaccine concepts

Response: The scientific precision, consistency of terminology, sentence structure and readability have been improved in the revised version of the manuscript and also the vaccine platform has been modified as multiepitope subunit vaccine.

Reviewer #2:

1. Can the predicted multi-epitope mRNA vaccine induce protective immunity against OROV in vivo?

Response: The current computational results indicate that the suggested vaccine may elicit protective immune responses. The incorporation of MHC class I, MHC class II, and B-cell epitopes could facilitate the probable stimulation of cytotoxic T lymphocytes, helper T cells, and antibody-secreting B cells. Furthermore, the predicted interactions with TLR2 and TLR4, the structural integrity noted throughout the 100 ns molecular dynamics simulation, and the immune simulation outcomes comprising predicted elevated antibody concentrations, IFN-γ synthesis, and memory cell proliferation—conclusively endorse its prospective immunogenicity. Nonetheless, these computational results do not validate that the vaccine will confer protective immunity against OROV in-vivo. The protective effectiveness must be determined using experimental methods involving recombinant expression and purification of the vaccine, succeeded by investigations on immunogenicity, safety, dose optimization, and viral challenges in an appropriate animal model. Such investigations ought to evaluate the generation of neutralizing antibodies, T-cell activity, cytokine profiles, viral load, clinical manifestations, survival rates, and tissue pathology. Additionally, these limitations have been added at the end of the discussion section.

2. How accurate are immunoinformatics-based epitope predictions compared with experimentally validated epitopes

Response: Immunoinformatics-driven epitope prediction tools offer a dependable and affordable method for ranking prospective vaccine candidates; yet, they cannot entirely replace experimental verification. The majority of modern predictive algorithms are constructed and refined utilizing extensive databases of experimentally confirmed epitopes, allowing them to discern potential antigenic areas with satisfactory predicted accuracy. However, in the future this will be confirmed by experimental validation and has been also added in the discussion section.

3. Why was disulfide engineering not performed to improve the structural stability of the vaccine construct?

Response: In the revised manuscript, the disulfide engineering has been performed.

4. Which discontinuous B-cell epitopes are present on the 3D structure of the vaccine construct, and how might they contribute to humoral immunity?

Response: The analysis of discontinuous B-cell epitopes was already part of the methodology. This analysis is actually the same thing as structural B-cell epitopes, which were discussed about in the 3.10 section of the materials and method along with an image and more information about the epitopes.

5. Why was the adjuvant placed at the N-terminal position instead of the C-terminal position in the vaccine construct?

Response: This has been mentioned at the methodology of the vaccine construction as “The adjuvant was deliberately placed at the N-terminal region, as this configuration is frequently utilised in multi-epitope vaccine development and is confirmed by immunoinformatics research. Positioning the adjuvant at the N-terminus enables its first translation and maintains its structural differentiation from the subsequent epitope sections, thus promoting effective identification by pattern recognition receptors (PRRs), including Toll-like receptors, contingent upon the chosen adjuvant. This orientation is anticipated to augment the primary innate immune activation, hence fostering more robust adaptive immunological reactions. Furthermore, the positioning of the N-terminal, in conjunction with a suitable stiff linker (such as EAAAK), aids in reducing structural disruption between the adjuvant and the epitope sequences, hence maintaining the autonomous folding and biological efficacy of each element. This design approach has been extensively utilised in earlier computational multi-epitope vaccine research due to its tendency to enhance structural stability and immunogenicity.”

6. Why was molecular docking specifically performed with TLR4 receptor?

Response: We would like to clarify that molecular docking was not performed exclusively with TLR4. In our study, the vaccine construct was docked with both TLR2 (PDB ID: 2Z7X) and TLR4 (PDB ID: 3FXI) to comprehensively evaluate its potential interactions with innate immune receptors. TLR2 and TLR4 were selected because they are key pattern recognition receptors (PRRs) involved in the initiation of innate immune responses through the recognition of pathogen-associated molecular patterns (PAMPs). Activation of these receptors triggers downstream signaling pathways that stimulate the production of pro-inflammatory cytokines and promote the development of adaptive immunity. Evaluating interactions with both receptors therefore provides a broader assessment of the vaccine candidate's potential immunostimulatory properties.

7. How do different untranslated regions (UTRs) influence mRNA stability and translation efficiency?

Response: We would like to clarify that, after the revision of the manuscript, the suggested design is uniformly characterized as a multi-epitope subunit vaccine instead of an mRNA vaccine. Consequently, the formulation and enhancement of mRNA-targeted regulatory components, such as 5′ and 3′ untranslated regions (UTRs), have been excluded from the vaccine development approach outlined in this research.

8. What dosage and delivery system would be optimal for this mRNA vaccine candidate?

Response: First, we would want to clarify that the proposed design, after manuscript modification, is consistently defined as a recombinant multi-epitope subunit vaccine, not an mRNA vaccine. Therefore, mRNA specific delivery methods, such as lipid nanoparticles, are not directly suitable for the present vaccine candidate. The delivery system has been added in the discussion of the revised manuscript as “For a recombinant subunit vaccine, computer analysis alone cannot be used to identify reliably the appropriate dose and delivery formulation. Based on standard procedures for recombinant protein vaccines, the purified vaccine design might be first assessed using intramuscular or subcutaneous administration with an appropriate authorized adjuvant or delivery system. Possible formulations include aluminium-based adjuvants, oil-in-water emulsions or biodegradable nanoparticle-based delivery methods. These formulations may improve the stability of the antigen, its uptake by antigen-presenting cells, and the generation of both humoral and cellular immune responses.”

9. Why was a full 100 ns Molecular Dynamics (MD) simulation not performed to validate the stability of the vaccine–TLR complexes?

Response: We have implemented a classical molecular dynamics simulation of the vaccine and vaccine–TLR4 complex in GROMACS within 100 ns, and for that a new section has been added in the revised manuscript. The trajectory was assessed using solvent-accessible surface area, hydrogen-bond analyses, radius of gyration, and RMSD and RMSF.

10. Could classical MD simulation using GROMACS or AMBER provide more realistic dynamic behavior of the vaccine construct compared with Normal Mode Analysis?

Response: Classical molecular dynamics simulation evaluates time-dependent atomic behavior under an explicitly defined force field and simulated physiological environment, whereas normal mode analysis provides a simplified assessment of collective molecular motions around an energy-minimized structure. Using GROMACS, we conducted a classical MD simulation of the vaccine–TLR4 complex in 100 ns to address this concern. Throughout the trajectory, this simulation allowed us to investigate structural deviation, residue-level flexibility, compactness, solvent exposure, and hydrogen-bond behavior. The results indicated that the complex maintained a structural profile that was relatively stable throughout the simulation.

11. How would RMSD, RMSF, radius of gyration (Rg), SASA, and hydrogen bond analyses from MD simulations support the structural stability of the vaccine complex?

Response: The vaccine–TLR4 complex maintained relatively limited overall structural deviation, as evidenced by the RMSD analysis, with values ranging from approximately 0.4 to 1.0 nm during the 100 ns simulation. The apo, in contrast, exhibited a larger degree of structural variation. The majority of the complex's residues exhibited minimal fluctuations, while the larger movements were restricted to specific regions rather than extending throughout the entire structure, as indicated by the RMSF analysis. This suggests that the complex maintained its overall structural organization, despite the localized flexibility. The complex's overall compactness was maintained without substantial unfolding or structural expansion, as evidenced by the relatively constant Rg profile. In the same vein, the SASA profile's stability indicated that the solvent exposure remained consistent throughout the trajectory. The presence of interactions during the simulation was further demonstrated by hydrogen-bond analysis, which corroborates the persistent molecular association between the vaccine and TLR4.

Combined, these analyses suggest that the vaccine–TLR4 complex maintained overall conformational stability, compactness, controlled residue flexibility, consistent solvent exposure, and persistent molecular interactions throughout the 100 ns simulation. The revised Results and Discussion sections now include these findings.

12. Can MM-PBSA/MM-GBSA calculations from MD trajectories provide more accurate binding free energy estimations than docking scores alone?

Response: We agree that MM-PBSA/MM-GBSA calculations that are conducted using representative frames extracted from an equilibrated molecular dynamics trajectory provide a more comprehensive estimation of binding free energy than docking scores alone, as they account for conformational changes that occur during the simulation. However, the MM-GBSA module, which is integrated into the HawkDock server, was employed to estimate the binding free energy in the current study. This module calculates the binding free energy by analyzing the docked protein–protein complex, rather than MD trajectory snapshots. This method has been extensively employed to evaluate protein–protein interactions and offers a more comprehensive evaluation of binding affinity than docking scores alone by integrating molecular mechanics and solvation energy terms. Additionally, we conducted a 100 ns classical molecular dynamics simulation in GROMACS to independently assess the structural stability of the vaccine and vaccine–TLR4 complex through RMSD, RMSF, Rg, SASA, and hydrogen-bond analyses, and we supplemented the HawkDock MM-GBSA analysis. Together, these complementary analyses offer computational support for the vaccine and vaccine–TLR4 complex's favorable interaction and stability. Additionally, we have added the lack of trajectory based MMGBSA/MMPBSA as a limitation and discussed that these will be conducted in future.

13. How can clinical and epidemiological findings on Oropouche virus infections support the real-world relevance and translational potential of the proposed vaccine candidate?

Response: The real-world relevance and translational potential of the proposed vaccine candidate have been added in the conclusion of the revised manuscript as “Overall, the favorable computational findings improve the proposed vaccine candidate's translational potential. The favorable physicochemical features, together with successful probable codon optimization and in-silico cloning, suggest the viability of recombinant production for further experimental studies. Although these findings need to be validated in-vitro and in-vivo, they provide compelling evidence for developing this multi-epitope subunit vaccine as a possible preventive candidate against Oropouche virus.”

Attachment Submitted filename: Response_to_Reviewers_auresp_2.docx

# Peer review: Decision Letter 2

**DOI:** 10.1371/journal.pone.0360186.r005

**Authors:** Shahina Akter

23 Sep 2026

An Immunoinformatics-driven Multiepitope Subunit Vaccine Targeting Oropouche Virus: Molecular docking, Immune and Dynamics Simulation Approaches

PONE-D-26-06233R2

Dear Dr. Ahmad Abdullah Mahdeen,

We’re pleased to inform you that your manuscript has been judged scientifically suitable for publication and will be formally accepted for publication once it meets all outstanding technical requirements.

Within one week, you’ll receive an e-mail detailing the required amendments. When these have been addressed, you’ll receive a formal acceptance letter and your manuscript will be scheduled for publication.

An invoice will be generated when your article is formally accepted. Please note, if your institution has a publishing partnership with PLOS and your article meets the relevant criteria, all or part of your publication costs will be covered. Please make sure your user information is up-to-date by logging into Editorial Manager at Editorial Manager® and clicking the ‘Update My Information' link at the top of the page. For questions related to billing, please contact billing support.

If your institution or institutions have a press office, please notify them about your upcoming paper to help maximize its impact. If they’ll be preparing press materials, please inform our press team as soon as possible -- no later than 48 hours after receiving the formal acceptance. Your manuscript will remain under strict press embargo until 2 pm Eastern Time on the date of publication. For more information, please contact onepress@plos.org.

Kind regards,

Shahina Akter, Ph.D.

Academic Editor

PLOS One

Additional Editor Comments (optional):

We sincerely thank Reviewer 1 for the thorough and constructive assessment. We are grateful for the recognition that the manuscript has improved considerably and that the major concern regarding the vaccine platform definition has been fully resolved. We will carefully address all remaining points, including tempering docking interpretations, correcting virological terminology, clarifying population coverage and predicted immune responses, and refining scientific wording. We are pleased that the manuscript will be acceptable for publication.

Shahina Akter, PhD

Academic Editor

Reviewers' comments:

Reviewer's Responses to Questions

**Comments to the Author**

Reviewer #1: (No Response)

Reviewer #2: All comments have been addressed

**********

2. Is the manuscript technically sound, and do the data support the conclusions?

Reviewer #1: Partly

Reviewer #2: No

**********

3. Has the statistical analysis been performed appropriately and rigorously?

Reviewer #1: Yes

Reviewer #2: (No Response)

**********

4. Have the authors made all data underlying the findings in their manuscript fully available?

The PLOS Data policy

Reviewer #1: Yes

Reviewer #2: Yes

**********

5. Is the manuscript presented in an intelligible fashion and written in standard English?

Reviewer #1: Yes

Reviewer #2: Yes

**********

Reviewer #1: Thank you for the thorough revisions and for the detailed responses to the previous review. The manuscript has improved considerably. In particular, the major concern regarding the definition of the vaccine platform has now been fully resolved. The construct is now correctly described as a multi‑epitope subunit vaccine, consistent with the recombinant protein expression strategy used throughout the study.

I also appreciate the addition of the 100 ns molecular dynamics simulation and the clearer acknowledgement of the limitations associated with the computational analyses. These updates strengthen the methodological clarity of the work. Most of the earlier concerns have therefore been satisfactorily addressed. The remaining points are more focused and should be straightforward to resolve without altering the overall design of the study.

1. Interpretation of docking results

The Abstract still refers to the TLR2 and TLR4 docking scores as “binding affinities” and suggests that they indicate “robust innate immune activation.” Docking scores should not be interpreted as experimentally determined affinities, nor do they demonstrate receptor activation. I recommend stating simply that the docking analysis predicted favourable interactions between the construct and TLR2/TLR4, without drawing functional conclusions.

2. Description of OROV replication

There are a few virological inaccuracies in the Introduction. Oropouche virus is a negative‑sense RNA virus, and its RNA‑dependent RNA polymerase transcribes and replicates RNA, not DNA. Please correct these statements and review the manuscript for similar errors.

3. Population coverage interpretation

The IEDB population coverage tool estimates the proportion of individuals predicted to present one or more epitopes based on HLA allele frequencies. It does not predict protection or vaccine efficacy. Phrases such as “effectively safeguarded” or “100% global coverage” should be revised to “predicted HLA population coverage” to avoid overinterpretation.

4. Interpretation of TLR2/TLR4 docking

Although the wording has improved, predicted interaction with TLR2/TLR4 should not be equated with immunostimulatory activity. A predicted interaction does not demonstrate receptor activation or downstream signalling. Please ensure this distinction is clear throughout the manuscript.

5. Rationale for selecting TLR2 and TLR4

The justification for choosing TLR2 and TLR4 remains general. If there is evidence that the vaccine construct or the 50S ribosomal protein adjuvant may interact with these receptors, it should be cited. Otherwise, the docking analysis should be presented as exploratory.

6. Positioning of the adjuvant

The explanation for placing the adjuvant at the N‑terminus includes statements that appear speculative. It would be more appropriate to frame this arrangement as a design choice consistent with previous multi‑epitope vaccine studies, with the EAAAK linker providing structural separation, rather than as an established immunological advantage.

7. Interpretation of molecular dynamics results

The addition of the 100 ns GROMACS simulation is a valuable improvement. The RMSD, RMSF, Rg, SASA, and hydrogen‑bond analyses provide useful insight into the behaviour of the modelled complex. Interpretation should remain within the scope of MD: stability of the complex under simulation conditions supports structural persistence, but does not confirm biological binding or receptor activation.

8. MM‑GBSA analysis

It is helpful that the manuscript now clearly states that the HawkDock MM‑GBSA calculation was performed on the docked complex rather than MD trajectory frames, and that this limitation is acknowledged. This is acceptable as long as the analysis is not presented as equivalent to trajectory‑based free‑energy calculations.

9. Predicted versus demonstrated immune responses

Some statements still imply that the construct has been shown to enhance humoral or cellular immunity. No experimental immunogenicity data were generated in this study. Please ensure that all such statements are framed as predicted responses based on epitope selection and immune simulation, rather than demonstrated effects.

10. Language and scientific wording

A final careful read is recommended to refine scientific wording and avoid expressions that overstate the biological significance of computational results (e.g., “probable standard binding affinities,” “protective proficiency,” “safeguarded the population”).

Overall, the authors have responded well to the major concerns raised in the previous review, and the manuscript is now substantially improved. The remaining points are minor and focused on clarity and interpretation. Addressing them will ensure that the conclusions remain appropriately aligned with what can be supported by an entirely computational vaccine‑design study.

Reviewer #2: (No Response)

**********

what does this mean?). If published, this will include your full peer review and any attached files.

If you choose “no”, your identity will remain anonymous but your review may still be made public.

**Do you want your identity to be public for this peer review?**  For information about this choice, including consent withdrawal, please see our Privacy Policy

Reviewer #1: No

Reviewer #2: No

**********

# Editor report: Acceptance letter

**DOI:** 10.1371/journal.pone.0360186.r006

**Authors:** Shahina Akter

PONE-D-26-06233R2

PLOS One

Dear Dr. Mahdeen,

I'm pleased to inform you that your manuscript has been deemed suitable for publication in PLOS One. Congratulations! Your manuscript is now being handed over to our production team.

At this stage, our production department will prepare your paper for publication. This includes ensuring the following:

* All references, tables, and figures are properly cited

* All relevant supporting information is included in the manuscript submission,

* There are no issues that prevent the paper from being properly typeset

You will receive further instructions from the production team, including instructions on how to review your proof when it is ready. Please keep in mind that we are working through a large volume of accepted articles, so please give us a few days to review your paper and let you know the next and final steps.

Lastly, if your institution or institutions have a press office, please let them know about your upcoming paper now to help maximize its impact. If they'll be preparing press materials, please inform our press team within the next 48 hours. Your manuscript will remain under strict press embargo until 2 pm Eastern Time on the date of publication. For more information, please contact onepress@plos.org.

You will receive an invoice from PLOS for your publication fee after your manuscript has reached the completed accept phase. If you receive an email requesting payment before acceptance or for any other service, this may be a phishing scheme. Learn how to identify phishing emails and protect your accounts at https://explore.plos.org/phishing.

If we can help with anything else, please email us at customercare@plos.org.

Thank you for submitting your work to PLOS One and supporting open access.

Kind regards,

PLOS One Editorial Office Staff

on behalf of

Dr. Shahina Akter

Academic Editor

PLOS One
